# Semantic Agency Patterns Signal Depressive Experiences: Evidence From Postpartum Communication on Social Media

**DOI:** 10.1155/da/6485997

**Published:** 2026-02-08

**Authors:** Marta Witkowska, Marta Beneda, Magdalena Formanowicz, Magda Leszko, Selen Arslan, Jan Nikadon, Joachim Kowalski, Tomaso Erseghe, Caterina Suitner

**Affiliations:** ^1^ Center for Research on Social Relations, SWPS University, Warsaw, Poland, swps.pl; ^2^ Department of Psychology, New York University Abu Dhabi, Abu Dhabi, UAE, nyu.edu; ^3^ Department of Information Engineering, University of Padua, Padua, Italy, unipd.it; ^4^ Department of Cognitive Science, Nicolaus Copernicus University, Toruń, Poland, umk.pl; ^5^ Institute of Psychology, Polish Academy of Sciences, Warsaw, Poland, pan.pl; ^6^ Department of Developmental Psychology and Socialisation, University of Padua, Padua, Italy, unipd.it

**Keywords:** agency, depression, linguistic markers, postpartum, social media

## Abstract

Depression‐related symptoms, such as loss of motivation and diminished interest in activities, correspond to loss of agency. Given recent evidence that agency (or its lack) can be reliably detected in language, we investigated how linguistic manifestations of agency relate to depressive experiences. In two studies, we explored whether semantic agency can serve as a novel marker of depressive experiences within the context of postpartum. We analyzed data from Twitter (Study 1, *N* = 17,664 tweets) and Reddit (Study 2, *N* = 3033 posts), using three complementary approaches: machine learning‐based topic detection, analysis of established linguistic markers of depression, and expert coding of depressive experiences. Across both studies, reduced semantic agency consistently emerged as a reliable indicator of depressive features. Posts discussing individuals’ depressive experiences in the postpartum period exhibited lower levels of semantic agency; semantic agency within posts was negatively correlated with established linguistic markers of depression; and semantic agency was negatively linked to depressive experiences as coded by experts. These findings highlight the potential of semantic analysis for mental health applications, suggesting that agency‐based markers could enrich existing linguistic frameworks examining psychological distress. While this research is at an early stage, future validation could clarify whether such markers might enhance the sensitivity of language‐based screening tools for identifying individuals in need of mental health support.

“[Depression] makes me completely incapable of doing things. When I’m at my worst I can barely drag myself out of bed. My concentration is affected, I can’t hold everyday conversations or complete everyday tasks. Even getting dressed feels like a challenge” (patient suffering from depression; after [1]). Such a profound sense of inability and powerlessness, which often leaves individuals almost entirely unable to act, is a common experience for people suffering from clinical depression. Indeed, fatigue or loss of energy, together with a markedly diminished interest or pleasure in all, or almost all, activities are two critical diagnostic symptoms of major depressive disorder, according to the Diagnostic and Statistical Manual of Mental Disorders [2]. This study aims to investigate whether experiences that could be potentially indicative of such symptoms are linked to specific patterns of language use, thereby contributing to the body of research on linguistic markers of depressive features^1^.

In diagnosis, depression is described with symptoms such as powerlessness, lack of motivation, lack of interest in activities that an individual used to enjoy, and, eventually, actual withdrawal from activities [2, 3]. When thinking about these symptoms through the prism of social cognition, they all come down to agency, one of the fundamental dimensions of social perception [4] and the most important dimension of self‐perception [5]. As a psychological dimension, agency refers to the capacity to attempt to achieve one’s goals and to plan and execute one’s actions [6]. Therefore, depression appears to coincide with the lack or loss of agency, and the link between these two concepts has received significant attention in the literature. In the classical model of depression, Beck [7] noted that depression fosters a biased perception of the self as passive rather than active. Relatedly, patients suffering from depression use internal attributions less often when explaining positive life events than nondepressed individuals [8]. Lower sense of agency has been associated with a higher risk of depression in both men and women [9] and with high suicidal ideation [10, 11]. Moreover, the lack of agentic themes in one’s fictional narratives has been found to mediate the relationship between one’s depressive symptoms and one’s negative emotional tone in written language [12]. In another narrative study, the frequency of agentic themes increased with the time spent in psychotherapy and was related to improvements in participants’ mental health [13].

While the above evidence concerns clinical samples, some support for the link between agency and the most common depressive symptom—low mood—can be derived from general population research. In a study, in which individuals operated fitness equipment accompanied by music, those who passively listened to the music reported lower mood compared to those who were granted “musical agency,” that is, the ability to modulate the music through their movements [14]. Similarly, in an experimental paradigm in which groups were taking turns singing and listening, participants’ mood improved after taking the agentic role (i.e., singing) and worsened after taking the non‐agentic one (i.e., listening; [15]). The sense of agency has also been linked to hope, with individuals who experience higher levels of this positive emotion setting a higher number of personal goals, and being more likely to meet such goals (see [16, 17]). Generally, positive mood appears to increase people’s life satisfaction by activating their sense of agency [18]. Moreover, the human brain seems to automatically pair positive mood with action: accompanying positive mood with stimuli that primed action and negative mood with stimuli that primed inaction increased performance in a cognitive task, as compared to the condition with the opposite mood‐stimuli pairings [19]. Relatedly, in a study on consumer choice, individuals who were subjected to a sadness induction procedure indicated a relative preference for a passive‐framed product over an active‐framed product [20].

In summary, the link between agency and depression, or low mood, has been observed across studies using different methodologies and conducted in a variety of contexts. This research aims to build upon these findings and provide preliminary evidence for the agency‐depression link in language. Despite strong theoretical foundations, agency as a linguistic marker of depressive experiences has received very little attention, with only one set of studies having assessed one aspect of agentic speech, the use of active (vs. passive) voice [21]. Our goal is to advance this research further by providing a comprehensive investigation of semantic aspects of agency, including of how these relate to existing well‐known markers of depressive features.

In the age of social media, where individuals produce as well as consume vast amounts of textual data as part of their daily routine, understanding how language can signal, and therefore aid the detection of depressive features has become a critical area of scientific investigation. Extensive research has identified several linguistic markers of depressive symptoms as well as correlates of the disorder (e.g., [22]). In the current investigation, we focused on those that are the most well‐established and have been validated through meta‐analytic procedures (e.g., [23]).

The first key marker of depressive features is the use of positive and negative emotion words; specifically, depressed individuals have been found to use more negative than positive emotion words (e.g., [24, 25]). While this pattern has been observed across diverse methodologies and samples—both offline and online—there has been some criticism regarding the validity of the marker. Specifically, concerns have been raised, both conceptual and empirical, about relying solely on simple word‐counting methods [26, 27]. Kross et al. [26] found that the frequency with which people use emotion words on Facebook neither predicts nor is predicted by their self‐reported emotional state, unlike human ratings of emotionality, which proved to be a reliable predictor [26]. Similarly, counting emotion words in participants’ everyday spoken language was found ineffective in predicting their daily mood changes [27]. Conceptually, assessing emotional expression, particularly in the context of social media, might be unreliable as any such expression may be modulated by individuals wanting to project a specific public image (see [26]). Moreover, longitudinal data suggest that the association between variability in the use of negative emotion words and depression severity is context‐dependent, as it appears to vary across different social media platforms. Specifically, on Facebook, temporal variance in the expression of negative emotion words was positively associated with depression severity, whereas on Twitter, the relationship between the two constructs was found to be negative [28]. Relatedly, the meta‐analysis conducted by Tølbøll [23] revealed that while the average effect size of the correlation between the use of negative emotion words and depression was significant, it might have been influenced by a publication bias.

In addition to the above, we believe that there is one more, previously overlooked, point of criticism concerning emotion words as markers of depression that should be highlighted. The cognitive view of depression proposes that while negative mood is a salient outward symptom of the condition, its root cause lies in idiosyncratic cognition—specifically, in pervasive negative beliefs about the self, the world, and the future [7, 29]. An individual might experience negative emotions without being depressed, especially when such emotions are evoked by situational factors rather than stemming from a biased perception of reality. To accurately distinguish depression from one’s temporary negative emotional state in cross‐sectional data, such as text samples on social media, it seems crucial to complement emotional markers of depression with cognitive ones. Namely, signals that are less vulnerable to contextual influences and, as such, might better capture the protracted nature of a depressive state. The use of first‐person pronouns seems to be one of the key indicators of this kind.

The prevalence of first‐person singular pronouns (i.e., “I‐words”) in one’s language has been suggested to reflect the tendency to self‐focus and internalize problems, two behavioral hallmarks of depression (e.g., [30]). The relationship between “I‐words” and depression has been found to be stable and universal, unaffected by demographic variables or publication bias [30]. However, “I‐words” have also been linked to other psychological outcomes, such as narcissism and neuroticism (e.g., [31]). Importantly, Tackman and colleagues [32] found that controlling for neuroticism diminished the link between depression and first‐person pronoun use, suggesting that the link could be largely explained by the overall relationship between “I‐words” and one’s general tendency towards negative emotionality.

Given these complexities, we argue that it is imperative to further expand the list of linguistic markers associated with depressive features, particularly those capturing its core cognitive aspects, in order to improve the sensitivity and accuracy of depression detection tools. This paper aims to provide an initial contribution to this effort.

As mentioned above, depression‐related symptoms such as loss of motivation and interest in activities can be seen as indicative of a diminished sense of agency [33]. This link between (low) agency and depression suggests that linguistic expressions of agency could serve as valuable markers of depressive symptoms. In this way, we integrate cognitive theories of depression—particularly Beck’s model of maladaptive self‐schemas [7, 29]—with linguistic analysis by translating the theoretical construct of diminished agency into a measurable semantic feature of language.

Although different aspects of language have been identified to signal agency (e.g., [34]), in this research we want to focus on semantic content, as most established linguistic markers of depression are also semantic in nature. Semantic agency reflects the extent to which individuals explicitly communicate agentic content through words associated with action, control, or intentionality [35, 36]. This approach is grounded in broader psycholinguistic research showing that semantic patterns in language provide meaningful insights into psychological states [37]. Efforts to evaluate and validate semantic manifestations of agency have already been underway for some time. For example, research on social perception has shown that words and phrases denoting agency are more frequently used to describe social categories stereotypically perceived as high in agency (e.g., men) compared to those perceived as low in agency (e.g., women; [38]). Additionally, a meta‐analysis on the behavioral effects of word priming found that exposure to goal‐related words increases engagement in subsequent goal‐oriented behavior [39]. Further, the use of semantic agency has been found to reflect gender‐related stereotypes in job advertisements for female‐ and male‐dominated occupations ([35], Study 4) and to influence perceptions of agentic traits and values in the descriptions of feminist campaigns [40]. It has also been observed to correlate with self‐report measures of agency ([35], Study 3). Finally, prior studies indicate that semantic agency constitutes a valid proxy of one’s sense of agency. Spontaneous use of agentic language correlates with self‐reported agency [41], and experimentally inducing agentic language has been shown to increase individuals’ reported sense of agency [42]. Taken together, these findings support semantic agency as a useful, though necessarily partial, linguistic indicator of underlying psychological agency.

Earlier approaches to measuring semantic agency relied primarily on dictionary‐based methods. Pietraszkiewicz et al. [35] and Nicolas et al. [43] developed validated lexicons of agentic terms (e.g., active verbs, achievement‐oriented adjectives), scoring texts based on the frequency of these words normalized by total word count. These tools demonstrated strong reliability and convergent validity, correlating with competence ratings and stereotype evaluations. However, their main limitation lies in their inability to account for linguistic context: words are treated as equally agentic regardless of usage, and phenomena such as polysemy or negation can lead to misclassification.

A novel approach to measuring semantic agency that builds on dictionary‐based methods while addressing their limitations is BERTAgent, a Python package developed by Nikadon et al. [36] BERTAgent is a transformer‐based language model fine‐tuned on textual data previously coded by human raters for agency. Extensive empirical validations have shown that BERTAgent aligns more closely with human judgments and outperforms dictionary‐based methods on tests of convergent and discriminant validity. It surpasses simple word‐counting techniques by accounting for the semantic context in which words are used, correcting for polysemy and negation. Moreover, BERTAgent is able to assess variations in both the intensity and directionality of agency. In other words, it distinguishes between lower (“This is a car, it runs on gas”) and higher (“This is Jane, she runs for office”) levels of agency, as well as between positive (“Striving to achieve my goals”) and negative (“Struggling to achieve my goals”) expressions of agency. As a result, BERTAgent offers a comprehensive assessment of semantic agency. However, it also has limitations: it is more complex and less transparent than lexicon‐based approaches, and its performance depends on the quality and representativeness of the training data.

The aim of this research is to examine semantic agency as a potential novel linguistic marker of depressive experiences. In line with prior work on linguistic markers of depressive symptoms, our investigation begins with analyzing language use on social media, which provides an unparalleled source of naturalistic language data. At this early stage, our analyses focus on self‐reported depressive experiences rather than clinically verified diagnoses. This approach enables us to explore semantic agency as a potential marker in a large‐scale, ecologically valid context, while also laying the groundwork for future, more costly studies involving clinically diagnosed populations. Across two independent studies, we analyze communication on Twitter (now “X,” Study 1) and Reddit (Study 2) to assess the links between agentic language and depressive experiences in the context of postpartum depression (the rationale for this focus is outlined below). Each study involves three analytical stages, using distinct methodological approaches.

The first stage verifies whether the data contain clusters of social media posts that discuss depression and those that do not, and evaluates whether the semantic agency of these clusters varies depending on the degree to which they are centered on depression. This step involves topic detection, an unsupervised machine learning method used to uncover latent topics in large text corpora by identifying common themes (e.g., word and phrase patterns). The second stage maps semantic agency in social media posts onto established linguistic markers of depressive symptoms, specifically the use of emotion words and “I‐words.” Finally, the third stage assesses whether semantic agency uniquely predicts depressive experiences, as coded by experts in a sample of posts.

Overall, we hypothesise that, across the two studies, semantic agency will be: less prevalent in depression‐related vs. depression‐unrelated clusters of posts (H1), negatively associated with the use of established linguistic markers of depression (H2), and negatively associated with the presence of self‐reported depressive experiences in a post as coded by experts (H3).

In this research, we focus on social media content containing hashtags or keywords related to the postpartum period, a topic predominantly discussed by women. This focus is motivated by findings that women tend to report a lower sense of personal influence and agency compared to men, which may contribute to the onset and persistence of depression [44]. Furthermore, women, particularly new mothers, are twice as likely as the general population to experience both mild and severe depressive symptoms (e.g., [45, 46]). In fact, it is estimated that up to 20% of women in Western contexts experience a depressive episode, commonly referred to as postpartum depression, following childbirth (e.g., [47, 48]). Many women affected by postpartum depression are also known to turn to online platforms to share their experiences, partly because such spaces offer a sense of community and reduced stigma (e.g., [49, 50]). However, since not all (new) mothers experience depressive symptoms, our language samples are also likely to provide a control group for statistical comparison. After all, the term “postpartum” encompasses a wide range of experiences, including physical, emotional, and relational changes and challenges that vary greatly between individuals (e.g., [51]). Posts might cover topics such as hair loss, weight gain, dietary choices, questions about child development or breastfeeding, or the joy of bonding with one’s newborn. Consequently, social media content related to the postpartum period is likely to display substantial variability in expressed agency.

## 1. Study 1 Method

This study investigates our hypotheses by analysing Twitter posts. While Twitter is primarily used for information consumption, it also functions as a platform for sharing information and building relationships [52]. Previous studies have successfully used Twitter data to examine linguistic markers of depression (e.g., [53]; for a review, see [54]). Additionally, research suggests that Twitter data can not only aid in detecting depression but also forecast its onset and progression [55]. Thus, we chose Twitter as the platform for our initial analyses. Notably, the 280‐character limit on tweets may constrain the amount of information shared in a single post, presenting a potential challenge. However, tweets still meet the minimum threshold of 25 words per text unit necessary for yielding reliable results in automated linguistic analyses ([56], p. 171). Furthermore, the option to create anonymous accounts may encourage users to disclose more personal information.

### 1.1. Data Collection

We used Twitter’s API V2 under an Academic License (November 2022) to collect tweets. Python was chosen as the programming language and the Tweepy library was employed to gather original tweets related to the topic of “postpartum” and/or containing the hashtag “#postpartum.” By original tweets, we mean user‐generated posts, as opposed to retweets, which are reposts of existing content. To ensure temporal balance in the dataset, data collection was conducted systematically, focusing on original tweets from 2021, targeting 50 tweets per day. All collected tweets were in English, with no geographical restrictions, allowing for a global representation. As a result of this process, a total of 17,664 tweets were gathered.

When analysing publicly available data, such as social media content, Institutional Review Boards grant exemptions from review. However, ethical considerations, particularly those concerning user privacy, remain a priority. To protect user privacy in our study, we followed the approach of Kwon and Park [57] by refraining from citing actual posts. Instead, we present paraphrased examples of original content, preserving the core message while safeguarding users’ privacy throughout the manuscript^2^.

Finally, given the large sample size, our interpretation of results—particularly for analyses involving the full dataset—will be guided by effect sizes rather than *p*‐values. In the absence of field‐specific benchmarks for effect sizes, we adhere to Cohen’s [58] guidelines, where Pearson’s *r* values of 0.10, 0.30, and 0.50 represent small, medium, and large effects, respectively.

### 1.2. Procedures and Measures

#### 1.2.1. Semantic Agency

We calculated the semantic agency score for each post using the BERTAgent algorithm [36], which quantifies the direction and level of agency expressed in a text. Scores range from −100 to 100, with negative scores indicating low agency and positive scores indicating high agency. For example, the sentence “I had the overwhelming feeling of sinking deeper and deeper” scored −46.64, representing low agency, whereas “Months into my postpartum journey, I ran my first kilometer, and I couldn’t be prouder!” scored 56.14, representing high agency. Neutral content that does not reflect either low or high agency would score close to zero. For instance, the sentence “For how long do they generally advise having a postpartum doula?” received a score of 0.08. The final score for each post was calculated as the average of the sentence‐level scores within that post.

#### 1.2.2. Topic Detection and Analysis

We used the BERTopic tool [59]^3^ to perform topic detection, enabling the identification of prominent themes within the data. BERTopic is a topic detection library based on BERT (Bidirectional Encoder Representations from Transformers; [60]), a transformer‐based machine learning technique for natural language processing. Once the algorithm^4^ identifies initial clusters (topics) in the data, it then employs an outlier reduction strategy^5^ based on “embeddings.” Using cosine similarity between an outlier text’s embedding and the topics’ embeddings, the algorithm assigns outliers to the closest topic. To streamline the process and reduce redundant computation, we used pre‐computed embeddings.

To understand the primary focus of each topic, we conducted a qualitative analysis of a randomly selected sample (10%) of posts for each topic. Based on their content, we created a label and description of each topic (see Table 1). Finally, to quantitatively assess the extent to which each topic was related to depression, we calculated a Term Similarity Score for the word “depression.” This score quantifies the degree of semantic association between the selected term and the dominant words within each topic. The similarity score ranges from 0 to 1, with higher scores indicating a stronger semantic relationship between the term and the topic. Specifically, we calculated cosine similarity [61] between the embedding of the selected term (“depression”) and the embeddings of the top 10 words that defined each topic. Cosine similarity is a measure that captures the angular distance between two vectors in a high‐dimensional space and is widely used to quantify the similarity between semantic vectors. All in all, the resulting Term Similarity Score reflected the extent to which each topic was conceptually aligned with the term “depression,” offering insight into which clusters of posts are most relevant to discussions surrounding this subject.

**Table 1:** Topics on Twitter with sample content and descriptive statistics.

Topic name	Representative words	Content summary	Tweet example	*N*	Semantic agency			Similarity score with “depression”
					*M*	Me	SD	
Depression: Advocacy	Depression postpartum psychosis woman symptom mental risk disorder experience baby	This topic includes discussions around mental health challenges, such as postpartum depression and psychosis, alongside advocacy for improved mental health services and sharing of information about these conditions. Although the content addresses sensitive and impactful subjects, only a small portion of the tweets reflect personal experiences, with even fewer offering present‐day accounts	Speak openly about your emotions and thoughts. Postpartum mental health challenges can sometimes worsen into psychosis, which is really concerning. I hope you’ll never have to go through anything like that	1549	−10.01	−9.97	14.80	0.87
Depression: Articles and others’ stories	Depression postpartum woman mother help suffer download mental baby therapy	The tweets feature quotes from clinical discussions on postpartum conditions (often including download links), excerpts from personal stories about mental and physical challenges faced during the postpartum period, and expression of personal opinions and experiences. Many posts highlight the need for support and the value of therapy. The majority of tweets are neutral in terms of sentiment, as they reference others’ stories	This is so intense. I’m mad at her, but I understand her perspective because I experienced bad postpartum depression after my daughter’s birth. I’m watching a show about it named NC	1118	−7.82	−7.29	17.67	0.87
Depression: Need for information	Depression talk help postpartum need know suffer people woman mom	These tweets address the emotional challenges and depressive experiences that can occur postpartum, emphasizing the value of sharing information. Many act as calls to action, offering support, guidance, and reassurance	I truly believe men should take mandatory courses on recognizing postpartum depression, anxiety, and rage, as well as how to support and cope with these. Women are pleading for more help. Mothers desperately need men to gain this understanding	324	−6.37	−6.39	13.86	0.77
Depression: Personal experiences	Depression postpartum feel like real go know people think talk	Tweets within this topic focus on postpartum depression, sharing both personal and others’ stories that detail symptoms, treatments, and the critical role of community support. Many tweets link to articles, providing additional information. A key theme is normalizing the conversation around mental health	I’m over saying I’m fine while knowing it’s a lie. Postpartum depression is tough, and it’s maddening to keep it hidden since people treat you as fragile instead of being helpful	972	−11.70	−11.93	15.15	0.77
Depression and anxiety	Anxiety postpartum fear feel depression symptom help bad mom have	This topic explores the anxiety and fears surrounding the postpartum period and parenthood. Authors express their emotions and the need for support. Concerns about health risks, particularly those related to postpartum recovery and COVID, are also commonly expressed in these posts	Postpartum recovery is hard, and I’m finding it difficult to feel motivated for anything. The anxiety and sense of being overwhelmed are constant. 6 days into a cleanse and focusing on prayer, I’m feeling a bit more like myself. Taking steps toward self‐love	367	−11.49	−11.63	15.12	0.70
Depression: Information broadcasting	Mental health depression support maternal help study postpartum care dr	Tweets within this topic focus on generic information about postpartum challenges, particularly postpartum depression. This includes updates on relevant research, social initiatives, news articles, and other sources	In 2016, research showed that postpartum depression impacted 38% of moms from minority communities, compared to 13%–19% of other moms. It’s okay to ask for help—postpartum doulas are there to support you and your little one	474	3.80	4.60	15.13	0.69
Hormonal changes	Hormone cry rage emotion postpartum emotional reason bitch like hormonal	The topic focuses on the hormonal changes and emotional volatility during the postpartum period, emphasizing their impact on a mother’s mental and emotional well‐being. Many tweets link to scientific research and news articles that explore these issues in greater detail	Having low progesterone may result in symptoms like PMS, repeated miscarriages, PPD, or even increase the risk of cancers such as breast or uterine. Thankfully, treatment is often possible. #hormonalhealth #infertilityawareness	316	−9.27	−9.59	14.46	0.58
Health complications	Black woman death pregnancy die blood risk postpartum maternal hemorrhage	Tweets within this topic address serious health risks associated with childbirth, particularly complications like maternal hemorrhage. These discussions encompass personal accounts of complications during and after delivery, mental health struggles, and broader concerns related to maternal safety racial disparities in healthcare. While some posts are neutral, others express deep emotional distress	In the U.S., the postpartum period is critical, with 40% of maternal deaths occurring in the first 6 weeks and almost 20% happening between 6 weeks and a year later. Paid leave is essential to support mothers’ recovery	708	−2.36	−2.80	14.54	0.51
Hair loss	Hair loss postpartum hairless grow growth fall shed bald thin	Tweets under this topic discuss the challenges of postpartum hair loss a common issue many new mothers face. These posts often feature pictures of women experiencing this problem, sharing their mixed emotions (often using emojis), or seeking advice on hair care and recovery. Some also provide tips for managing hair loss after childbirth	Postpartum hair loss is tough enough with all these hormones, but my son just pulled out some of my hair and attempted to snack on it	961	−8.74	−10.01	12.81	0.49
Pelvic floor recovery	Postpartum pelvic recovery floor day like go know baby week	This topic focuses on the physical recovery after childbirth, particularly concerning pelvic floor health. While most posts convey a neutral sentiment, the discussions often address serious and impactful issues	Pelvic floor issues are often shrouded in shame, preventing women from seeking treatment. Postpartum care and rehabilitation should be financially supported for everyone	904	−1.57	−1.23	14.40	0.47
Hospital experiences	Care postpartum nurse baby hospital birth delivery newborn pregnant woman	The tweets within this topic explore interactions and experiences in hospital settings, highlighting the medical support needed during the postpartum period. Many of these posts reference news articles	Maternal leukocyte levels recorded during labor may signal early infections after birth and potential neonatal complications	1234	3.53	4.57	13.66	0.47
Body image	Body week look postpartum photo month like love skin good	This topic is centered around the physical and emotional changes in the postpartum period, with a strong focus on body image. Tweets range from sharing practical tips, like using cooling pads for pain relief, to deeply personal reflections on struggles with mental health	I’ve been exercising for about a month now, and wow, I feel amazing! I can’t wait to see how much more progress my body makes if I stick with it this month. I’m months postpartum and finally starting to feel like myself again	879	0.47	1.69	12.80	0.45
Emotional support seeking	Feel postpartum like shit go hard bad baby hate suck	This topic features candid posts that often serve as an outlet for emotions related to current physical and mental states. Some express feelings of inadequacy and exhaustion—whether personal or concerning public figures or characters in movies and series. Many posts seek emotional support or a sense of solidarity	Postpartum recovery is so hard, especially when everyone expects you to bounce back instantly. It’s like they think giving birth is nothing and recovery happens overnight. All they care about is meeting the baby	1044	−7.27	−7.90	16.62	0.45
Healthcare coverage	Coverage medical extend state expand health year bill care maternal	This topic includes tweets addressing various aspects of medical support and coverage for postpartum care. Tweets describe efforts to extend healthcare benefits and strengthen support systems for new mothers, including legislative initiatives. They tend to have a neutral tone, as they are primarily informational and impersonal	Postpartum Medicaid extension is still on the table as part of the Medicaid Technical Amendments bill SB. Our analysis sheds light on its importance and the outcomes it could bring	537	12.19	12.94	13.65	0.45
Institutional and systemic support	Support care postpartum help need parent work new service birth	This topic includes posts discussing and/or linking articles exploring the various forms of support new parents need, including healthcare services, community resources, as well parental leave	The postpartum period can be incredibly exhausting and overwhelming, especially for mothers recovering from Cesarean births. Check out our blog for insights into SSI risks linked to CDs and tips for postpartum care for both mom and baby!	902	9.57	11.21	13.85	0.45
Self‐care and gifts for new mothers	Postpartum gift mom day baby like new box go get	The tweets within this topic feature advertisements for gifts and gift baskets for new mothers as well as links to postpartum care resources and questions or calls for taking care of one’s mental and physical well‐being. The tone is generally neutral or positive	Caring for your mind and body is the ultimate gift to yourself. Find out how Annie has been focusing on self‐care these days	1575	1.49	2.60	13.85	0.45
Breastfeeding	Postpartum month pregnancy pregnant breastfeed woman baby period week time	This topic focuses on breastfeeding challenges, time management, and its effects on the menstrual cycle. Many tweets share information, link to articles, or promote related products	Whoever said breastfeeding was simple wasn’t being honest. I thought my baby would latch immediately when I became a mom. Well, that didn’t happen. I now understand why postpartum depression can be so common. This is tough, but staying strong is the only way forward	2238	−0.16	0.25	14.50	0.45
Fitness	Workout exercise fitness gym yoga run photo postpartum body week	This topic includes discussions about resuming fitness routines after childbirth, personal stories of adapting workout routines, motivational accounts from new mothers, and critiques of celebrities rushing to regain pre‐pregnancy bodies. Most posts convey a neutral sentiment, focusing on informative and supportive content	Do you experience back discomfort or feel disconnected from your core muscles? These classes are designed to provide tailored support, helping you lay the groundwork for effective strength training	363	12.44	11.97	17.10	0.44
Weight loss	Weight lose lbs pound week gain postpartum prepregnancy day body	This topic includes tweets discussing weight changes during the postpartum period. Posts share personal experiences with losing or managing weight after childbirth, addressing both the physical and emotional aspects of returning to pre‐pregnancy weight. The discussions also feature tips and supportive advice	Just under 3 weeks postpartum, and I’m already 15 pounds lighter than before pregnancy. It’s amazing what our bodies can do!	454	−0.57	0.09	13.90	0.41
Postpartum recovery	Waist belt belly wear underwear clothe trainer band postpartum fit	The tweets within this topic focus on physical recovery after childbirth, highlighting the use of postpartum belts and waist trainers to help new mothers regain their pre‐pregnancy body shape. Many are advertisements, while others share personal recommendations for specific products designed to aid postpartum recovery	Designed for postpartum mothers, the Angelica Recovery garment helps ease pain and speed up recovery, ensuring moms can regain their strength to care for their newborn. It stands apart by being solely dedicated to recovery	745	1.33	2.75	10.70	0.35

#### 1.2.3. Established Linguistic Markers of Depression

Linguistic Inquiry and Word Count (LIWC; [62]) is a tool that provides indices for established linguistic markers of depression: the percentage of negative emotion words, positive emotion words, and first‐person singular pronouns in a text (e.g., [25, 63]). LIWC operates by categorising words based on its dictionaries and calculating their frequencies as percentages of all relevant words in the text. The “negative emotions” dictionary includes 618 words, such as “worry,” “sad,” “cry,” and “angry.” The “positive emotions” dictionary contains 337 words, such as “good,” “love,” “happy,” and “hope.” Lastly, the “I‐words” dictionary comprises six words, such as “I,” “me,” “my,” and “myself,” which form 74 individual entries (i.e., word stems and phrases). In this study, we used LIWC 22 [64] to generate the three variables of interest using these dictionaries, with scores ranging from 0 to 100, representing the proportion of relevant words in each collected post.

#### 1.2.4. Depressive Experiences as Coded by Experts

Users’ depressive experiences were identified through an expert coding process in which two psychologists independently assessed a randomly selected subsample of 2000 posts. The coders used the two core PHQ‐2 items (“feeling down, depressed, or hopeless” and “little interest or pleasure”; [65]) as conceptual guidelines for content analysis. Coders indicated the presence (“yes” = 1) or absence (“no” = 0) of these features in users’ posts. To ensure reliability, any disagreements between the two coders were resolved by a third psychologist with clinical expertise.

According to the coding guidelines, the experts indicated the presence of depressive experiences only when posts clearly reflected the individual’s current and personal experience of depressive features. Posts discussing other aspects of the postpartum experience or mentioning depression without referencing one’s own recent experiences were excluded. For instance, posts speculating about others’ mental health (e.g., “Postpartum disorders are far more common than acknowledged and are largely misunderstood. Britney might have suffered due to this…”), describing the past (e.g., “I barely recall my son’s earliest days, as postpartum depression clouded my mind. I’m forever grateful for my partner, my GP, and my friends for looking out for me back then.”), or discussing anticipated depression (e.g., “Just imagining going through postpartum depression is bringing me down emotionally.”) were excluded, as were posts authored by professionals offering psychoeducational content (e.g., “Specialists explain ways to access support for postpartum depression, anxiety, and stress after giving birth.”).

An example of a post meeting the coding criteria is: “Feeling drained but so anxious that relaxing feels impossible. Is anyone else going through this? Maybe it’s part of postpartum depression?”

## 2. Study 1 Results

### 2.1. Stage 1: Detection and Analysis of Depression‐Related and Depression‐Unrelated Clusters of Data

**Figure 1:**
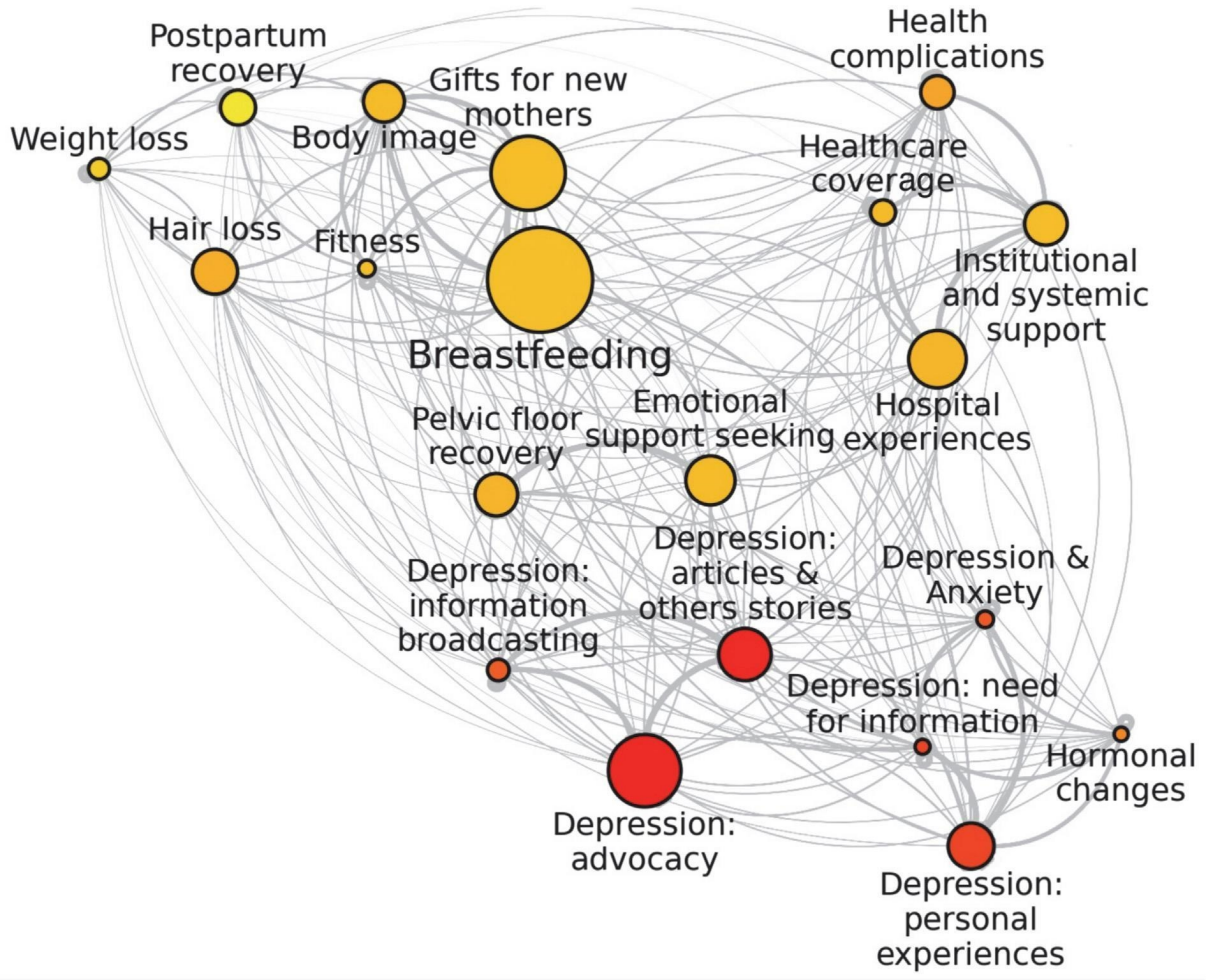
Community network of topics on Twitter. *Note:* The size of the nodes represents the size of topics (number of posts), while the color palette reflects the levels of similarity to “depression,” with darker colors indicating higher similarity scores.

The topic detection process identified 20 distinct topics related to various aspects of the postpartum period, as visualised in Figure 1. These topics varied in size, with the largest one (2238 posts) focusing on *breastfeeding* and the smallest (316 posts) on *hormonal changes*.

**Figure 2:**
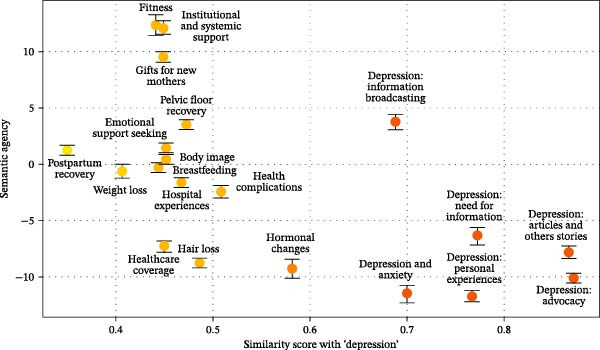
Mapping semantic agency to the similarity score with “depression” across topics on Twitter. *Note*: The color palette indicates levels of similarity to “depression,” with darker shades representing higher similarity scores. The error bars denote standard errors.

The qualitative analysis of posts within topics (see Table 1) revealed that many topics centered on emotional or mental health issues, such as depression and anxiety, highlighting the psychological challenges of the postpartum period. Notably—and perhaps unsurprisingly, given the nature of Twitter (“X”) as a social media platform—many tweets were information‐driven, often linking to scientific articles, news reports, or referencing ongoing events and political debates. The specificity of topics varied: some were focused on particular concerns such as hair loss, gift ideas, or fitness routines (e.g., *Hair loss*, *Gifts for new mothers*, *Fitness*), while others encompassed broader themes related to lifestyle changes and experiences of maternity (e.g., *Body image*, *Depression: Information broadcasting*, *Depression: Articles and others’ stories*). Personal narratives featured prominently in certain topics (e.g., *Breastfeeding*, *Depression: Personal experiences*, *Depression and anxiety*), though the majority of posts advocated for policy change or enhanced support systems, often linking to external resources (e.g., *Institutional and systemic support*, *Healthcare coverage*, *Depression: Information broadcasting*). Posts indicative of users’ depressive experiences appeared across multiple topics but were most concentrated in the following two: *Depression: Personal experiences* and *Depression and anxiety*, which focused specifically on real‐time, personal accounts. This diversity in the content suggested that the dataset was well‐suited for analysis, capturing a broad spectrum of content both related and unrelated to depressive experiences. Further support for this comes from the variability in the similarity scores with the term “depression,” which ranged from 0.35 to 0.87 across topics (see Table 1 and Figure 2).

For each topic, we calculated descriptive statistics for semantic agency (see Table 1 and Figure 2), revealing considerable variation, with some topics displaying notably high or low levels of semantic agency and others remaining relatively neutral. To evaluate the relationship between semantic agency and the degree to which topics centered on depression (H1), we calculated the correlation between the similarity score for the term “depression” and semantic agency across topics. This analysis revealed a strong negative correlation, *r*(18) = −0.60, *p* = 0.005, as visualized in Figure 2. These findings indicate that topics more closely related to the term “depression” tend to exhibit lower levels of semantic agency.

In the above analysis, we adopted a methodology consistent with prior research, which identifies depressive features through the use of the term “depression” and other similar terms within text [66, 67]. However, we consider the evidence obtained through this approach as only preliminary, given the inherent limitations of identifying depressive features based solely on semantic proximity. Specifically, the similarity score reflects how closely a topic aligns semantically with the term “depression,” but it does not account for sentiment, leaving the tone of the message undetermined. Consequently, the score may encompass mentions of depression in neutral or even positive contexts, such as quotations, reflections on recovery and post‐depression growth, or even jokes. Furthermore, this method cannot differentiate between posts authored by professionals, family members of those suffering from depression, and those personally affected. To address these limitations, we proceeded to subsequent stages of analysis.

### 2.2. Stage 2: Mapping Semantic Agency to Established Linguistic Markers of Depression

Following Huson’s [68] recommendation for correlational analyses with zero‐clustered data, we conducted Pearson correlations to examine the relationship between semantic agency and established linguistic markers of depressive features^6^. To ensure robust standard errors, tweets were nested within authors using the *survey* package in R [69]. The results (see Table 2) revealed that semantic agency was significantly associated with all established depression markers in the expected directions, with effect sizes ranging from small to medium. The strongest correlation observed was a negative association between semantic agency and negative emotion words, indicating that posts with lower semantic agency were more likely to express aversive emotional states. Similarly, posts with lower semantic agency tended to include fewer positive emotion words, though this relationship was weaker. The weakest correlation was observed between semantic agency and the use of “I‐words.” To explore this further, we examined the correlations between “I‐pronouns” and both positive and negative emotion words, finding them negligible, in contrast to prior research (e.g., [25]). This pattern suggests that “I‐words” in our data were somewhat neutral, lacking a clear emotional tone or connection to agency. This could be attributed to Twitter’s nature as a broadcasting platform, where “I” can be used in a variety of contexts—from sharing personal information to offering expertise or linking external resources—many of which are emotionally neutral or ambivalent.

**Table 2:** Descriptive statistics for all linguistic markers and bivariate correlations between them (Twitter).

	*M*	SD	1	2	3
(1) Semantic agency	−1.98	15.90	—	—	—
(2) I‐words	4.66	6.29	−0.12 ^∗∗∗^	—	—
(3) Positive emotion words	3.50	5.06	0.23 ^∗∗∗^	−0.04 ^∗∗∗^	—
(4) Negative emotion words	4.53	7.01	−0.43 ^∗∗∗^	−0.02 ^∗∗^	−0.13 ^∗∗∗^

*Note:* ^∗∗^ *p* < 0.01;  ^∗∗∗^ *p* < 0.001; *p*‐values were adjusted using the Holm–Bonferroni method within each family of tests.

### 2.3. Stage 3: Mapping Semantic Agency to Depressive Experiences as Coded by Experts

The final set of analyses aimed to examine whether semantic agency contributes to predicting the odds of expert‐coded depressive experiences. Specifically, we tested whether including semantic agency in the model significantly improves its ability to predict depressive experiences, beyond the contribution of negative and positive emotion words as well as first‐person singular pronouns.

To address this, we conducted two separate logistic regression analyses. In both, the dependent variable was a binary indicator of whether depressive experiences were or were not inferred from a post. The predictors were the linguistic features of the post, that is, negative emotion words, positive emotion words, first‐person singular pronouns, and semantic agency. To evaluate the incremental contribution of semantic agency, we compared two models: a full model, which included all four linguistic features, and a reduced model, which excluded semantic agency. Robust standard errors were computed to account for potential non‐independence of posts written by the same author.

Table 3 summarizes the results of each model, showing how changes in the predictors are associated with the odds of a post being classified as reflecting depressive experiences. For each one‐unit increase in a predictor, the odds of a post being classified as depression‐related (vs. non‐related) change by the corresponding odds ratio (OR). We compared the two models by evaluating their −2 Log‐Likelihood (−2LL) statistics. The full model had a −2LL of 882.70 (df = 5), while the reduced model had a −2LL of 923.74 (df = 4). The difference between the models was statistically significant, *χ* ^2^(1) = 41.04, *p* < 0.001, indicating that the inclusion of semantic agency significantly improved the model’s fit and explained additional variance in classification of depression‐relatedness, beyond that explained by other linguistic features.

**Table 3:** Depression experiences as coded by experts regressed on linguistic markers (Twitter).

Predictor	Full model			Reduced model		
	*B* (SE)	OR (95% CI)	*p*‐Value	*B* (SE)	OR (95% CI)	*p*‐Value
(Intercept)	−3.86 (0.17)	—	<0.001	−3.73 (0.17)	—	<0.001
Negative emotion words	0.05 (0.01)	1.05 (1.03–1.07)	<0.001	0.08 (0.01)	1.08 (1.06–1.10)	<0.001
Positive emotion words	0.01 (0.02)	1.01 (0.98–1.05)	0.498	−0.01 (0.02)	0.99 (0.95–1.03)	0.577
I‐words	0.10 (0.01)	1.11 (1.08–1.13)	<0.001	0.11 (0.01)	1.12 (1.10–1.14)	<0.001
Semantic agency	−0.04 (0.01)	0.96 (0.95–0.97)	<0.001	—	—	—

*Note*: *B*, regression coefficient; *p*‐values are based on robust standard errors, accounting for clustering by author.

Abbreviations: 95% CI, 95% confidence interval for the odds ratio; OR, odds ratio; SE, standard error.

The results from the full model reveal that negative emotion words, first‐person singular pronouns, and semantic agency were all significant predictors of expert‐coded depressive experiences, while positive emotion words did not reach statistical significance. Specifically, higher frequencies of negative emotion words, first‐person pronouns, and lower semantic agency scores were associated with a greater likelihood of posts being classified as suggestive of depressive experiences. In the reduced model, where semantic agency was excluded, negative emotion words and first‐person singular pronouns remained significant predictors, with positive emotion words continuing to show no significant effect.

## 3. Study 2 Method

Social media platforms differ significantly in terms of user expectations, content, and user interactions. Twitter is a profile‐based “self‐media” platform, where messages are disseminated through a follower–followee system. In contrast, Reddit is a collaboration‐driven platform centered on users’ shared interests, where content takes precedence over personal connections [70]. Importantly, on Reddit, posts are organised within interest‐based subreddits that users subscribe to, fostering an online environment that can mitigate the influence of social desirability. This, in turn, encourages more genuine and less socially constrained engagement, including expressions of negative affect [71] and controversial opinions [72]. For these reasons, we chose to use Reddit data in Study 2 to capture a more authentic representation of user experiences. Additionally, Reddit’s lack of message‐length restrictions allows for longer texts, providing richer material for automated linguistic analysis using LIWC ([56], p. 171).

### 3.1. Data Collection

We collected Reddit data using the Pushshift API, accessed through the PRAW library (Python Reddit API Wrapper). The PRAW library provides a robust interface for efficiently scraping Reddit data based on specific subreddits, keywords, and time periods. As in Study 1, we selected posts containing the keyword “postpartum,” written in English and posted in 2021, with no geographical restrictions. During the quality control process, we identified and removed duplicate posts—original posts shared by the same users across multiple subreddits. While users may have duplicated posts to reach a broader audience or receive faster responses, such repetition is undesirable for analysis. After removing duplicates and performing necessary preprocessing, we were left with 3033 unique posts.

Expecting an even greater sensitivity of the data compared to Study 1, we again decided to paraphrase all social media content presented in this paper to ensure user privacy, while maintaining the essence of the original message.

### 3.2. Procedures and Measures

The procedures and measures were largely consistent with Study 1; this section highlights only the modifications.

#### 3.2.1. Topic Detection and Analysis

It is important to note Reddit posts were substantially longer (*M*_words_ = 247.97, SD_words_ = 297.72, Me_words_ = 162) than Twitter posts (*M*_words_ = 24.21, SD_words_ = 14.16, Me_words_ = 22). Longer texts often contain multiple topics, which can reduce the efficiency of BERTopic. To address this, we segmented each post into smaller, coherent units—sentences or short paragraphs—following recommendations by Jiang et al. [73]. This approach allowed for more focused topic modeling and enhanced BERTopic performance. The final dataset consisted of 44,025 textual elements.

As in Study 1, after data clustering, we conducted a qualitative analysis of posts representing each topic. For each topic, we randomly selected a minimum of 10% of posts in which the topic was dominant (i.e., most of the sentences within the post were assigned to that topic). Each selected post underwent a content analysis to understand, summarise, and categorise the thematic content related to postpartum experiences. Based on this process, we produced a summary of each topic’s content (see Table 4).

**Table 4:** Descriptive statistics for all linguistic markers and bivariate correlations between them (Reddit).

	*M*	SD	1	2	3
(1) Semantic agency	−5.76	9.08	—	—	—
(2) I‐words	8.51	3.92	−0.34 ^∗∗∗^	—	—
(3) Positive emotion words	3.58	4.03	0.26 ^∗∗∗^	−0.15 ^∗∗∗^	—
(4) Negative emotion words	2.83	2.51	−0.48 ^∗∗∗^	0.19 ^∗∗^	−0.14 ^∗∗∗^

^∗∗^ *p* < 0.01,  ^∗∗∗^ *p* < 0.001.

#### 3.2.2. Depressive Experiences as Coded by Experts

With Reddit posts averaging 10 times the word count of Twitter posts and a median word count seven times higher, coding Reddit posts benefited from the richer informational context available in each post. The greater level of detail in Reddit posts minimised ambiguity when categorising content. Consequently, we decided to code a sample of 500 posts, relying on the same coders as in Study 1. Given the nature of the coding process—assessing whether an individual’s post reflected depressive experiences based on their discourse—it was more appropriate to analyse the data at the post level rather than the sentence level, regardless of posts consisting of sentences with various levels of depression‐related content. This is because it is unlikely for someone to experience a negative mood or anhedonia when writing one sentence but not the next, even if the thematic content within the post shifts.

## 4. Study 2 Results

### 4.1. Stage 1: Detection and Analysis of Depression‐Related and Depression‐Unrelated Clusters of Data

**Figure 3:**
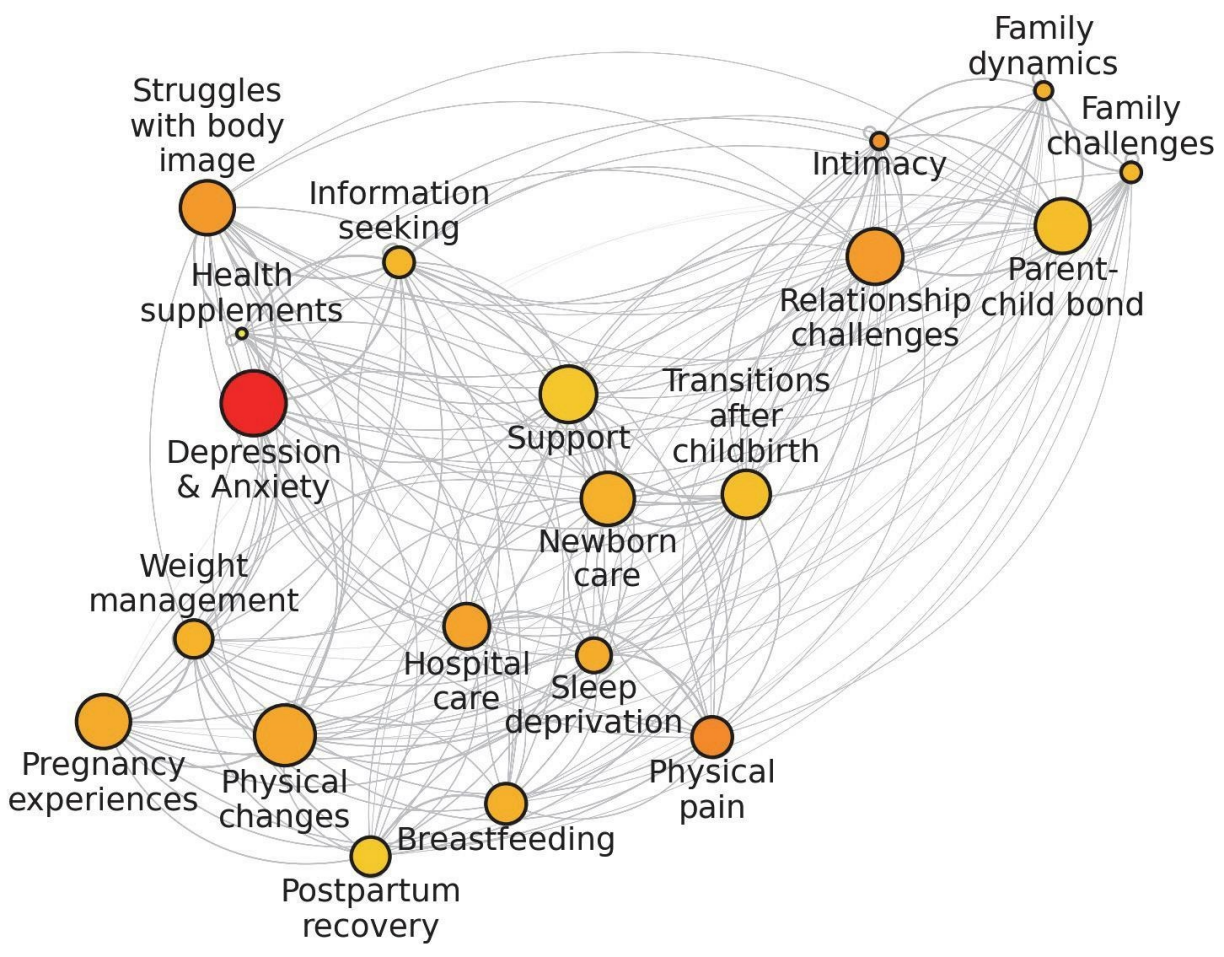
Community network of topics on Reddit. *Note:* The size of the nodes represents the size of topics (number of sentences), while the color palette reflects the levels of similarity to “depression,” with darker colors indicating higher similarity scores.

As in Study 1, the topic detection process identified 20 topics related to various aspects of the postpartum period, as visualised in Figure 3. These topics varied in size, with the largest—*Depression and anxiety*—comprising 3427 sentences, and the smallest—*Supplements*—comprising 568 sentences.

The qualitative analysis of topic content (see Table 5) revealed that, compared to tweets analysed in Study 1, Reddit posts included less information sharing and more in‐depth personal disclosures. There were fewer links to articles, reports, or references to ongoing events. This pattern aligns with expectations that Reddit, compared to Twitter, fosters a more personal and open communication environment [74, 75], where users are more inclined to share intimate or difficult experiences.

**Table 5:** Topics on Reddit with sample content and descriptive statistics.

Topic name	Representative words	Content summary	Post example	*N*	Semantic agency			Similarity score with “depression”
					*M*	Me	SD	
Depression and anxiety	Depression anxiety postpartum mental health feel like stress disorder go	This topic focuses on postpartum mental health issues, particularly depression and anxiety. Posts within this topic often include personal stories and requests for advice on managing mental health, and offer support from those who have faced similar challenges	Now that I’m 3 weeks postpartum, I’m overwhelmed with feelings of anxiety, stress, and worry. It’s almost like I’m grieving my former self. These heavy and disruptive thoughts feel like too much to bear. Do others feel similarly after childbirth?	3427	−16.36	−17.94	19.97	0.85
Physical pain	Pain section hurt painful tear preeclempsia feel week blood pressure	Posts within this topic focus on managing postpartum pain and health complications. They discuss coping with physical pain, recovering from childbirth‐related injuries, and dealing with long‐term health concerns. Posters share their experiences to warn others and offer advice on how to manage these challenges	1 week postpartum, and peeing remains extremely painful for me. I’ve been avoiding fluids due to how anxious I feel about using the toilet. Does anyone have tips to make this more bearable?	2122	−10.20	−10.37	14.92	0.51
Intimacy	Love sex relationship woman partner feel want drive find like	This topic included discussion centered on intimacy and relationship changes during the postpartum period, covering subjects such as sexual desire and romantic connections. Posters talk openly about the impact of childbirth on their partnerships, seeking support and offering perspectives	Almost a year postpartum, I’ve noticed a complete lack of romantic or sexual interest in my partner since my pregnancy began. I keep reminiscing about old relationships and the feelings they gave me. Does anyone else relate?	919	−4.80	−1.93	22.25	0.49
Struggles with body image	Feel like feeling know look cry hate think body want	Posts within this topic address dissatisfaction with postpartum body image, self‐esteem, and adjusting to physical changes after childbirth. Posters seek reassurance, share success stories, and offer advice to help others feel less isolated in their experiences	Postpartum depression and anxiety have been a struggle for me for months. I began running and going to the gym to lift myself out of it. Sometimes I bring my little one along in a jogging stroller. It’s tough, like pushing a car at times, but it’s been worth it—I’m feeling so much better now	2819	−17.32	−19.94	24.46	0.46
Relationship challenges	Husband say want time talk wife tell go friend marriage	Posts within this topic explore relationship challenges after childbirth, particularly focusing on communication and emotional connection. Posters frequently vent frustrations, seek advice on common issues such as reduced intimacy or improving communication, and share experiences of relationship strain	My husband reached out to my friends to ask them to look out for me since I’m always tired and avoiding intimacy or long talks. Am I being unfair, or should he just accept that postpartum comes with these challenges?	2936	−6.95	−6.68	21.80	0.46
Hospital care	Hospital nurse husband baby hour labor midwife come appointment care	This topic includes posts focused on experiences with hospital care during childbirth, including interactions with medical staff, decisions made during labor, and the overall birthing experience. Many posts feature personal labor stories, as well as discussions about healthcare providers and the care received	We’re relocating back home and preparing for the arrival of our first baby. I need advice on selecting an OB or hospital—any recommendations or insights on why one is better? Can we have a midwife accompany us? The pregnancy is considered low risk, but a c‐section might be necessary because of my size	2384	−2.16	−0.56	17.47	0.44
Physical changes	Postpartum week month baby birth period hormone day time like	This topic is centered on the body’s postpartum changes—hormonal, aesthetic, and physical. Posts cover recovery, hormonal shifts, the return of the menstrual cycle, and fertility. Posters share personal experiences or seek advice on navigating these adjustments	Breastfeeding moms, when did you get your first postpartum period? I’m 1 month out and think it could be happening soon, but it’s hard to tell	3194	−4.15	−2.92	16.18	0.43
Pregnancy experiences	Pregnancy pregnant week get birth exercise baby month start day	This topic is centered on pregnancy, focusing on stages, prenatal care, and preparations for the delivery. Posts often seek advice, share insights, or express concerns about staying physically and emotionally healthy during pregnancy	Can anyone else relate to feeling both beautiful and unattractive during pregnancy? I admire what my body is doing but also feel unappealing and not sexy at all. Postpartum scares me too, and I feel bad for even admitting that since it seems so minor compared to having a baby. My partner tries to help, but I’m still struggling emotionally. Is it just me?	2847	−2.00	−2.09	19.24	0.42
Sleep deprivation	Sleep night wake tired hour bed morning time day asleep	This topic consists of posts talking about postpartum sleep challenges, often reflecting the exhaustion and frustration of disrupted sleep patterns. Posters share strategies for better rest, seek advice, and discuss tips for managing sleep with a newborn	First‐time mom here, and we’re getting through the sleep regression this month, but it’s been overwhelming. Between a challenging birth, being isolated during lockdowns, family living at a distance, my husband’s crazy hours in film, and being the first in my friend group to have a child, I’m struggling a lot	1818	−10.28	−10.13	20.44	0.42
Family dynamics	Family sister husband brother friend live parent aunt want wife	Posts within this topic focus on family dynamics and relationships during the postpartum period, with posters discussing interactions with extended family and the complexities of maintaining healthy relationships	I just gave birth to my in‐laws’ first grandchild a few weeks ago, and now they’re pressuring us to come over for Christmas, even though they’re Muslim and don’t celebrate it. They’ve been quite pushy about their religious beliefs, and during their last visit, I felt disrespected as a mom	957	−6.74	−4.86	19.00	0.41
Breastfeeding	Breastfeed pump breast milk formula feed bottle supply exclusive nipple	This topic includes posts related to breastfeeding, exploring both the practical and emotional aspects of infant feeding. Discussions involve seeking advice on challenges such as milk supply, sharing personal experiences, and expressing the diverse emotions tied to feeding choices	I could use some encouragement! I’m a month postpartum and haven’t been able to exclusively breastfeed, but I really want to. I’m determined to continue my breastfeeding journey. Am I being unrealistic for pushing so hard?	2140	−4.12	−2.74	18.96	0.41
Newborn care	Baby bear birth old month give newborn like want child	This topic is centered on early motherhood and newborn care, with posts exploring both the challenges and joys of the first weeks postpartum. Discussions include general parenting concerns, personal stories, and the emotions surrounding this significant life transition	Hi 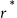 ! I’ve seen many posts here expressing concerns about handling the early days with a newborn. I think a lot of people don’t fully understand what life with a baby is like, and that fear of the unknown is completely normal. I’d like to share some insights about the reality of those first days, hoping it might help others feel more confident in their decisions	2778	−6.00	−4.10	19.56	0.40
Weight management	Weight eat lose gain lbs pound food meal diet healthy	This topic is focused on weight management during and after pregnancy, with posts addressing subjects such as weight gain, postpartum weight loss, and diet advice. Posters share body image challenges, success stories, and provide reassurance and support for those navigating struggles with eating or self‐image	I’m just over 4 weeks postpartum and struggling with thoughts about my weight. I’ve lost 20 pounds since delivery—mostly baby, placenta, and fluid—but I’m still considered overweight by BMI, and it’s tough to deal with. How did others manage to lose weight after having their little one?	1988	−3.71	−2.80	19.90	0.40
Information seeking	Hi think hello post happen ask aita hey question thought	Posts within this topic encompass a wide spectrum of postpartum issues, ranging from physical health to emotional well‐being. Posters share personal updates and discuss concerns, fostering a supportive space for diverse topics to be explored	Hi everyone! I’m new here and just found out I’ll be having a planned C‐section next week because of medical concerns. I’d love to hear your tips for managing the postpartum phase—it would mean a lot!	1606	−4.56	−1.19	20.56	0.39
Family challenges	Mil house home visit stay live work room want leave	Posts within this topic delve into household dynamics, focusing on visits from extended family or in‐laws. Common themes include seeking advice on setting boundaries, managing expectations, and coping with the stress such visits can cause	Ever since I brought my baby home a few weeks ago, people have been visiting constantly, making the days more draining and disrupting both my baby’s and my sleep. My in‐laws expect us to attend birthday parties at their homes and restaurants over an hour away, but as I’m breastfeeding, it’s hard. They don’t seem to get it and insist I start visiting them with the baby. To top it off, I’m getting my second COVID‐19 shot tomorrow, and they want to come over that night. Is it selfish to prioritize my recovery and say no?	1083	−3.95	−2.24	19.37	0.38
Parent–child bond	Mom parent daughter child kid mother want son dad old	This topic focuses on parental relationships, particularly between mothers and their children, emphasizing the significance of familial bonds and responsibilities. Posters often seek or share advice on parenting challenges, offering personal experiences that provide both guidance and encouragement	This pregnancy wasn’t planned—it really caught us off guard. I feel a mix of guilt because my daughter is still so young and excitement at the same time. My husband, surprisingly, handled the news really well!	2903	−6.24	−4.78	22.00	0.37
Transitions after childbirth	Week work go day pp month year time advance thank	Posts within this topic address the transitions following childbirth, including balancing work and family and managing emotions when leaving a child with other caregivers. Posters often share strategies and personal experiences to support others during this adjustment period	With twins on the way, we’re considering a postpartum doula to support us in the early weeks. This person would provide care, advice, and do a little tidying. Have you used a doula? What worked well, and was it worth the expense? Our goal is to get a couple of nights of decent sleep each week. We’re feeling overwhelmed and unsure what to expect when the twins arrive	2513	−1.62	−1.04	21.98	0.37
Support	Thank experience advice know story hope share tell appreciate expect	The topic includes posts discussing postpartum recovery—both physical and emotional—often expressing gratitude for shared advice and support. Subjects range from seeking reassurance to celebrating small victories, fostering a sense of shared experience and practical encouragement	What was your experience like during those initial postpartum weeks? What worked well for recovery and adapting to the big changes? Any advice you can offer would mean a lot—thanks so much!	2956	−4.56	−0.81	23.37	0.34
Postpartum recovery	Wear clothes pad underwear dress stretch fit papers skin size	This topic is centered on practical postpartum care, with posts discussing essentials like clothing, pads, and other necessities. Posters share experiences, recommend products, and offer advice, creating a supportive space to help navigate early motherhood with confidence	I’m a few weeks from delivery and completely unprepared in the clothing department for postpartum. Any ideas for affordable options?	2067	−5.27	−3.93	15.17	0.34
Health supplements	Nan calcium yada high nanny mg ionize strand Chinese calculator	Posts within this topic focus on postpartum wellness, covering supplement use, vitamins, and overall health maintenance to aid in recovery. Posters share advice on boosting well‐being and discuss strategies for maintaining health during the postpartum period	After giving birth, I’ve had swelling in my arms, hands, and lips. Has anyone gone through this? If so, are there any vitamins or supplements that helped you?	568	−8.49	−6.36	22.68	0.22

The thematic scope of posts varied widely. Some topics focused on specific challenges, such as managing postpartum pain (e.g., *Physical pain*), breastfeeding practices (e.g., *Breastfeeding*), or the use of recovery products (e.g., *Postpartum recovery*). Others addressed more general themes, such as lifestyle adjustments and mental health (e.g., *Depression and anxiety*, *Family dynamics*), encompassing a wide range of experiences. Posts related to mental health, including depression and anxiety, were primarily concentrated within the *Depression and anxiety* topic. However, mentions of depression also appeared in topics centered on other postpartum concerns, such as *Physical pain*, *Intimacy*, *Relationship challenges*, and *Struggles with body image*. This suggests that discussions of depressive experiences frequently intersect with broader postpartum challenges, reflecting the multifaceted nature of psychological distress during this period. The variability in topic focus highlights the thematic diversity of the dataset, which is further supported by the wide range of semantic similarity scores with the term “depression,” varying from 0.22 to 0.85 across topics (Table 5).

**Figure 4:**
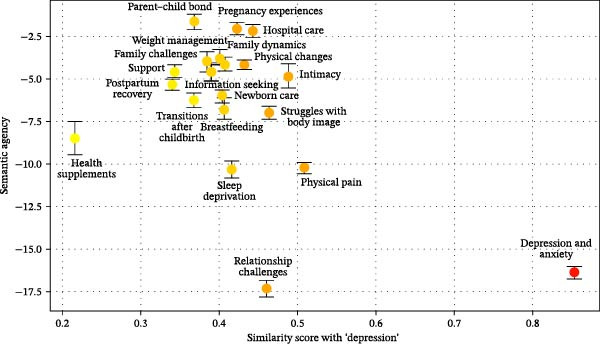
Mapping semantic agency to the similarity score with “depression” across topics on Reddit. *Note:* The color palette indicates levels of similarity to “depression,” with darker shades representing higher similarity scores. The error bars denote standard errors.

For each topic, we calculated descriptive statistics for semantic agency (see Table 5), revealing meaningful variation across topics, with some exhibiting high or low levels of semantic agency, and others remaining relatively neutral. To evaluate the link between semantic agency and topics’ depression‐centeredness (H1), we calculated correlations between semantic agency and the similarity score for the term “depression.” The analysis revealed a strong negative association, *r*(18) = −0.54, *p* = 0.014, as visualized in Figure 4. Topics related to “depression” displayed lower levels of semantic agency, compared to those unrelated to “depression,” replicating findings from Study 1. However, due to the same limitations discussed in Study 1, we implemented further, more restrictive analytical procedures to enhance the robustness of our results.

### 4.2. Stage 2: Mapping Semantic Agency to Established Linguistic Markers of Depression

As in Study 1, we conducted Pearson correlation analyses examining the relationships between semantic agency and established linguistic markers of depression, with posts nested within authors to obtain robust standard error estimates^7^. The results (see Table 4) revealed associations between semantic agency and all established depression markers in the anticipated directions, with effect sizes ranging from small to medium. Consistent with Study 1, the strongest correlation was a negative association between semantic agency and negative emotion words, while a medium‐sized correlation was observed with “I‐words.” The weakest correlation emerged with positive emotion words. In contrast to Twitter posts, the use of “I‐words” in Reddit posts correlated positively with negative emotion words and negatively with positive emotion words. This pattern aligns with Reddit’s function as a platform for personal expression, contrasting with Twitter’s more information‐broadcasting orientation. Given that the data were collected in the context of postpartum, the more intimate style of Reddit communication may have amplified the connection between self‐referential language and emotions experienced during this challenging time.

### 4.3. Stage 3: Mapping Semantic Agency to Depressive Experiences as Coded by Experts

As in Study 1, the final set of analyses examined whether semantic agency contributed to explaining the odds of expert‐coded depressive experiences. We conducted two separate logistic regression analyses, with a binary indicator of whether depressive experiences were inferred from a post as the dependant variable. Negative emotion words, positive emotion words, first‐person singular pronouns, and semantic agency were included as predictors. We compared a full model (including all predictors) with a reduced model (excluding semantic agency). Robust standard errors were computed to account for potential non‐independence of posts written by the same author.

Table 6 summarises the results of both models, showing how changes in each predictor were associated with the odds of a post being classified as reflecting depressive experiences. We compared the two models by evaluating their −2LL statistics. The full model had a −2LL of 337.50 (df = 5), while the reduced model had a −2LL of 351.98 (df = 4). The difference between the models was statistically significant, *χ^2^* (1) = 14.48, *p* < 0.001, indicating that the inclusion of semantic agency significantly improved the model’s fit and explained additional variance in the classification of depression‐relatedness, beyond that explained by other linguistic features.

**Table 6:** Depression experiences as coded by experts regressed on linguistic markers (Reddit).

Predictor	Full model			Reduced model		
	*B* (SE)	OR (95% CI)	*p*‐Value	*B* (SE)	OR (95% CI)	*p*‐Value
(Intercept)	−5.06 (0.52)	—	<0.001	−5.10 (0.53)	—	<0.001
Negative emotion words	0.23 (0.08)	1.26 (1.08–1.47)	0.002	0.33 (0.07)	1.39 (1.20–1.60)	<0.001
Positive emotion words	0.06 (0.03)	1.06 (1.01–1.11)	0.033	0.05 (0.03)	1.05 (0.99–1.11)	0.135
I‐words	0.19 (0.04)	1.21 (1.12–1.31)	<0.001	0.23 (0.04)	1.26 (1.16–1.36)	<0.001
Semantic agency	−0.07 (0.02)	0.93 (0.89–.97)	0.001	—	—	—

*Note: B*, regression coefficient; *p*‐values are based on robust standard errors, accounting for clustering by author.

Abbreviations: 95% CI, 95% confidence interval for the odds ratio; OR, odds ratio; SE, standard error.

Consistent with Study 1, the results from the full model revealed that negative emotion words, first‐person singular pronouns, and semantic agency were all significant predictors of expert‐coded depressive experiences. Unlike in Study 1, positive emotion words also emerged as a significant predictor, though in an unexpected direction. In the reduced model, negative emotion words and first‐person singular pronouns remained significant predictors, while positive emotion words no longer reached statistical significance (*p* = 0.135).

## 5. Discussion

This research explored the relationship between semantic agency and depressive experiences in the postpartum context using natural language data from two social media platforms: Twitter (Study 1) and Reddit (Study 2). Across both studies, the findings consistently supported our hypothesis that diminished semantic agency is a reliable indicator of depressive experiences. This conclusion is strengthened by the application of three distinct methodological approaches. First, machine learning‐based topic detection revealed that semantic agency was less prevalent in clusters of depression‐related posts compared to clusters of depression‐unrelated posts (H1). Second, a correlational analysis of established linguistic markers of depression demonstrated their associations with semantic agency in the expected directions (H2). Third, expert coding of depressive experiences indicated that semantic agency was negatively associated with coder‐identified depression‐related content in social media posts (H3). While all three methods are commonly employed in social media and mental health research (e.g., [28, 76]), each offers unique advantages. Topic detection and linguistic marker analysis are effective for identifying depression‐related content in large datasets, whereas human coding allows for a more nuanced and precise classification of depressive experiences, albeit limited to smaller data samples. Notably, prior research suggests that human coding of textual data can enhance the predictive accuracy of models beyond linguistic markers alone [77]. Overall, by combining these approaches, our multi‐method studies highlight the potential of semantic agency as a robust linguistic marker for detecting self‐reported depressive experiences in social media content.

Our research makes a broader contribution to understanding how language reflects psychological states. While the link between (low) agency and depression is well‐established in psychological literature, our findings extend this evidence by showing that these patterns can be effectively detected through linguistic analysis. Integrating cognitive theories of depression—which emphasize the role of distinctive thought patterns—with linguistic theories examining how these patterns manifest in communication, our findings underscore the importance of agency in understanding depression through language. In particular, by grounding semantic agency in Beck’s cognitive model, where a diminished sense of agency is central to depressive thinking, we show how a core theoretical construct can be translated into a measurable linguistic feature.

Extending the list of validated linguistic markers of depression addresses the need for a more comprehensive perspective on this complex disorder, in which cognitive, emotional, and behavioral processes interact to create a clinical picture that varies across individuals. Our findings revealed that the three linguistic markers—semantic agency, negative emotion words, and “I‐words”—have independent effects when included simultaneously in regression models predicting depressive experiences. This highlights their distinct but additive role as predictors, suggesting that each marker captures a unique facet of depressive features. These results underscore that integrating affective and cognitive linguistic markers provides a more comprehensive operationalisation of depressive experiences in line with cognitive theories, which view distorted thinking patterns and diminished agency as central to the disorder.

Linguistic analysis of social media data offers a nonintrusive way to identify psychological states in real time, making it a valuable tool for both research and clinical applications. As digital communication increasingly emerges as one of the dominant forms of interaction, identifying at‐risk individuals based on their social media language presents a promising avenue for prevention and early intervention efforts. Our findings suggest that semantic agency may constitute a relevant, yet previously overlooked, linguistic dimension in the context of depressive features. However, before it can be integrated into practical detection algorithms, further validation is needed. Future clinical research should test whether semantic agency reliably distinguishes individuals with clinical depression from those unaffected or in transient distress. Once validated, this construct could improve the sensitivity and theoretical grounding of existing language‐based models (e.g., [78, 79]), and aid in identifying individuals who may benefit from timely support, particularly among adolescents, who frequently use social networking platforms to discuss mental health [80].

### 5.1. Limitations and Future Directions

Despite the promising findings, several limitations of this research should be acknowledged. First, relying on social media data introduces inherent biases, as these samples may not fully represent the broader population. Social media users may differ from non‐users in factors such as the availability of social support or their willingness to share personal experiences online. Additionally, users of different social media platforms might vary systematically, as each platform fosters unique modes of communication and discussion topics. While our findings were largely consistent across Studies 1 and 2, small discrepancies likely reflect such sample variations. For instance, Reddit users appeared to disclose more personal information than Twitter users, a pattern consistent with previous research. Manikonda and colleagues (2018) found that during the #MeToo movement, Twitter primarily served to spread the message and express empathy towards victims, while Reddit was used to share personal stories. Similarly, Rohde et al. [75] found that intimacy‐related words were more prevalent in Reddit posts compared to Twitter posts in discussions about inflammatory bowel disease.

Our findings further suggest that communication contexts may influence not only the content but also the relevance of specific linguistic indicators. For example, in our Reddit data, “I‐words” were associated with both negative and positive emotion words, consistent with prior research linking them to emotional expression. Conversely, on Twitter, “I‐words” showed no relationship with emotion words, suggesting differences in how this linguistic cue is used pragmatically. Future studies should investigate the mechanisms underlying these contextual variations to better understand how platform‐specific dynamics shape linguistic patterns.

Second, our focus on depression in the postpartum period, a form of depression with unique contributing factors and manifestations [81], may limit the generalisability of the findings. Unlike other types of depression, postpartum depression is influenced by a complex interplay of hormonal changes, emotional challenges, and the stresses of new parenthood. Moreover, it is a condition that disproportionately affects women. Future research should investigate whether similar associations between semantic agency and depressive experiences emerge in other populations and contexts. Prior clinical literature indicates that men and women often exhibit different symptoms of depression due to cultural socialisation patterns, with men typically displaying more externalising symptoms, such as irritability or anger, and women showing more internalising symptoms like sadness or anxiety [82]. Thus, future studies should consider potential variation in symptom expression across gender and other social groups.

Third, our analyses were based on self‐reported depressive experiences in social media posts rather than clinically verified diagnoses. While this approach provides access to an unparalleled volume of ecologically valid, naturalistic language data, it also introduces ambiguity, as posts may reflect transient moods, rhetorical strategies, or broader cultural discourses rather than clinically significant disorders. While we made significant efforts to ensure methodological rigor, including the use of expert coding and established topic detection techniques, the potential for error remains, particularly when interpreting complex or ambiguous posts. Future research should aim to replicate our findings among individuals with verified clinical diagnoses to strengthen the validity and reliability of semantic agency as a marker of depressive features.

Furthermore, as with any psychological construct, agency can manifest in multiple ways—explicitly and implicitly, verbally and behaviorally. Semantic agency provides a valuable yet partial lens into this complexity. Its reliance on explicit language may underestimate psychological agency in cases of self‐censorship or overestimate it in contexts of strategic self‐presentation. Future research should adopt multi‐method and multi‐theoretical approaches to triangulate semantic agency with complementary behavioral, self‐report, and neurophysiological indicators.

Finally, although BERTAgent represents a major advancement over dictionary‐based measures of semantic agency by accounting for context, polysemy, and negation, it too has limitations. It is less transparent, depends on the representativeness of training data, and captures only explicit expressions of agency. While cultural and social biases are not unique to transformer‐based models—they also shaped earlier dictionary‐based approaches—these considerations highlight the need for cautious interpretation. Moreover, while our focus on semantic agency provides a systematic and validated approach, it necessarily narrows the scope of linguistic agency as a broader construct. Language can convey agency not only through semantic content but also through grammatical (e.g., verb use, transitivity, passive vs. active voice), discourse‐level (e.g., topic control, word order), and pragmatic (e.g., hedging, rhetorical emphasis) features. Each of these layers may reflect distinct or subtler facets of how individuals experience and communicate agency. By concentrating on semantic agency, our findings should therefore be interpreted as addressing one dimension of a multifaceted construct. Future research should systematically integrate semantic, grammatical, and pragmatic indicators to build a more comprehensive account of how agency manifests in language.

## 6. Conclusion

In conclusion, this research highlights semantic agency as a promising linguistic marker of depressive experiences, particularly in the postpartum period. By combining linguistic analysis with both traditional and machine learning‐based methods, we observed that reduced agency in language is consistently linked to different operationalisations of depressive experiences. These findings hold important implications for both theory and practice, offering new avenues for understanding and tackling depression in naturalistic, real‐world settings. As the field of digital mental health continues to advance, incorporating semantic agency analysis could strengthen our ability to detect and respond to the mental health needs of individuals suffering from depression more effectively.

## Funding

This research was funded by the OPUS 22 grant (2021/43/B/HS6/02819) awarded to Marta Witkowska from the Polish National Science Center. Magda Leszko’s contribution was supported by subsidies from the Ministry of Education and Science for maintaining and developing the didactic and research potential of the Psychology Institute at the SWPS University (9/2024). The contributions of Magdalena Formanowicz and Jan Nikadon were financed by the OPUS 19 grant (2020/37/B/HS6/02587) from the Polish National Science Center.

## Conflicts of Interest

The authors declare no conflicts of interest.

## Endnotes

1

2

3

4

5

6

7

## Supporting Information

Additional supporting information can be found online in the Supporting Information section.

## Supporting information


**Supporting Information** Distributional properties of the linguistic variables used in the correlation analyses are reported in the Supplementary Online Materials (Figures S1–S3 for Study 1; Figures S4–S6 for Study 2). Semantic agency showed an approximately symmetric, near‐normal distribution, whereas word‐count measures were highly skewed and zero‐inflated. To account for these distributional characteristics, we conducted robustness checks using Spearman rank‐order correlations with cluster‐robust standard errors; results were highly consistent with Pearson correlations (Tables S1–S2).

## Data Availability

The data used in this article are available in the OSF archive: https://osf.io/rcx8k/?view_only=16b7c89fc1634467ac93c5009039a7e1. Due to the sensitive nature of the textual data (i.e., original post content), data may be shared by the authors only upon obtaining appropriate approval from the IRB at the requester’s home institution. None of the reported studies were preregistered.

## Footnotes

1
^1^We use the term “depressive experiences” to denote self‐reported affective and cognitive states that share phenomenological features with clinical depression but whose diagnostic status remains unknown. Specifically, depressive experiences refer to subclinical or unverified manifestations of depressive features (e.g., sadness, hopelessness, reduced motivation, or self‐critical thoughts), for which it is not possible to determine whether they meet the formal diagnostic criteria for major depressive disorder. This distinction is particularly relevant in the context of social media data, where self‐reported depression‐like states are not subject to standardised clinical assessment, and information about symptom duration, intensity, or functional impairment is typically unavailable.

2
^2^To uphold a high level of objectivity in the paraphrases, we utilized ChatGPT 4o. The exact wording of the prompt is available in Supporting Information.

3
^3^ https://maartengr.github.io/BERTopic/.

4
^4^The parameters used in BERTopic were: n_neighbors in UMAP: 250; min_cluster_size in HDBSCAN: 80; diversity in topic model: 0.5.

5
^5^ https://maartengr.github.io/BERTopic/getting_started/outlier_reduction/outlier_reduction.html.

6
^6^Additional analyses examining the distributional properties of the linguistic variables and robustness checks using Spearman rank‐order correlations are reported in the Supplementary Online Materials (Supporting Information Figures S1–S3 and Table S1).

7
^7^Additional analyses examining the distributional properties of the linguistic variables and robustness checks using Spearman rank‐order correlations are reported in the Supplementary Online Materials (Supporting Information Figures S4–S6 and Table S2).

## References

[1] Slaby J. , Paskaleva A. , and Stephan A. , Enactive Emotion and Impaired Agency in Depression, Journal of Consciousness Studies. (2013) 20, no. 7-8, 33–55, https://janslaby.com/static/publications/SlabyPaskalevaStephan2013_JCS_EnactiveEmoDepression_Author_Proof.pdf.

[2] American Psychiatric Association , Diagnostic and Statistical Manual of Mental Disorders, 2013, 5th edition, American Psychiatric Publishing, 10.1176/appi.books.9780890425596.

[3] Burton C. , McKinstry B. , Szentagotai Tătar A. , Serrano-Blanco A. , Pagliari C. , and Wolters M. , Activity Monitoring in Patients With Depression: A Systematic Review, Journal of Affective Disorders. (2013) 145, no. 1, 21–28, 10.1016/j.jad.2012.07.001, 2-s2.0-84872843797. 22868056

[4] Abele A. E. and Wojciszke B. , Advances in Experimental Social Psychology, Advances in Experimental Social Psychology. (2014) 50, 195–255, 10.1016/B978-0-12-800284-1.00004-7, 2-s2.0-84904701434.

[5] Wojciszke B. , Baryla W. , Parzuchowski M. , Szymkow A. , and Abele A. E. , Self-Esteem is Dominated by Agentic Over Communal Information, European Journal of Social Psychology. (2011) 41, no. 5, 617–627, 10.1002/ejsp.791, 2-s2.0-79960747425.

[6] Bandura A. , Social Cognitive Theory: An Agentic Perspective, Annual Review of Psychology. (2001) 52, no. 1, 1–26, 10.1146/annurev.psych.52.1.1, 2-s2.0-0035227452. 11148297

[7] Beck A. T. , Depression: Clinical, Experimental and Theoretical Aspects, 1967, Harper and Row.

[8] Békés V. , Pessimistic Explanatory Style and Lack of Agency Characterise the Narratives of Depressive Patients, Psychiatria Hungarica. (2011) 26, no. 1, 36–45. 21502670

[9] Bandura A. , Toward a Psychology of Human Agency, Perspectives on Psychological Science. (2006) 1, no. 2, 164–180, 10.1111/j.1745-6916.2006.00011.x, 2-s2.0-84993790889. 26151469

[10] Bryan C. J. , Andreski S. R. , McNaughton-Cassill M. , and Osman A. , Agency is Associated With Decreased Emotional Distress and Suicidal Ideation in Military Personnel, Archives of Suicide Research. (2014) 18, no. 3, 241–250, 10.1080/13811118.2013.824836, 2-s2.0-84904681623. 24712868

[11] Hobbs M. and McLaren S. , The Interrelations of Agency, Depression, and Suicidal Ideation Among Older Adults, Suicide and Life-Threatening Behavior. (2009) 39, no. 2, 161–171, 10.1521/suli.2009.39.2.161, 2-s2.0-67649324417. 19527156

[12] Joo M. and Park S. W. , Depression is Associated With Negativity in TAT Narratives: The Mediating Role of Agency, Current Psychology. (2021) 40, no. 6, 3065–3072, 10.1007/s12144-019-00245-6, 2-s2.0-85064497779.

[13] Adler J. M. , Living Into the Story: Agency and Coherence in a Longitudinal Study of Narrative Identity Development and Mental Health Over the Course of Psychotherapy, Journal of Personality and Social Psychology. (2012) 102, no. 2, 367–389, 10.1037/a0025289, 2-s2.0-84859958145. 21910554

[14] Fritz T. H. , Halfpaap J. , Grahl S. , Kirkland A. , and Villringer A. , Musical Feedback During Exercise Machine Workout Enhances Mood, Frontiers in Psychology. (2013) 4, 10.3389/fpsyg.2013.00921, 2-s2.0-84891670454, 921. 24368905 PMC3857889

[15] Boyd M. , von Ranson K. M. , Whidden C. , and Frampton N. M. A. , Short-Term Effects of Group Singing Versus Listening on Mood and State Self-Esteem, Psychomusicology: Music, Mind, and Brain. (2020) 30, no. 4, 178–188, 10.1037/pmu0000266.

[16] Snyder C. R. , Harris C. , and Anderson J. R. , et al.The Will and the Ways: Development and Validation of an Individual-Differences Measure of Hope, Journal of Personality and Social Psychology. (1991) 60, no. 4, 570–585, 10.1037/0022-3514.60.4.570, 2-s2.0-17144470627. 2037968

[17] Snyder C. R. , Ilardi S. S. , Cheavens J. , Michael S. T. , Yamhure L. , and Sympson S. , The Role of Hope in Cognitive-Behavior Therapies, Cognitive Therapy and Research. (2000) 24, no. 6, 747–762, 10.1023/A:1005547730153, 2-s2.0-0033819498.

[18] Chang Y. P. , Algoe S. B. , and Chen L. H. , Affective Valence Signals Agency Within and Between Individuals, Emotion. (2017) 17, no. 2, 296–308, 10.1037/emo0000229, 2-s2.0-84987914585. 27642658

[19] Albarracin D. and Hart W. , Positive Mood + Action = Negative Mood + Inaction: Effects of General Action and Inaction Concepts on Decisions and Performance as a Function of Affect, Emotion. (2011) 11, no. 4, 951–957, 10.1037/a0024130, 2-s2.0-80052288350. 21859209 PMC3626447

[20] Rucker D. D. and Petty R. E. , Emotion Specificity and Consumer Behavior: Anger, Sadness, and Preference for Activity, Motivation and Emotion. (2004) 28, no. 1, 3–21, 10.1023/B:MOEM.0000027275.95071.82, 2-s2.0-3843115736.

[21] Simchon A. , Hadar B. , and Gilead M. , A Computational Text Analysis Investigation of the Relation between Personal and Linguistic Agency, Communications Psychology. (2023) 1, no. 1, 10.1038/s44271-023-00020-1, 20. 39242909 PMC11332215

[22] Loveys K. , Torrez J. , Fine A. , Moriarty G. , and Coppersmith G. , Cross-Cultural Differences in Language Markers of Depression Online, *Proceedings of the Fifth Workshop on Computational Linguistics and Clinical Psychology: From Keyboard to Clinic*, 2018, 78–87, 10.18653/v1/W18-0608.

[23] Tølbøll K. B. , Linguistic Features in Depression: A Meta-Analysis, Journal of Language Works - Sprogvidenskabeligt Studentertidsskrift. (2019) 4, no. 2, 39–59, https://tidsskrift.dk/lwo/article/view/117798.

[24] Rude G. , Gortner E. M. , and Pennebaker J. W. , Language use of Depressed and Depression-Vulnerable College Students, Cognition and Emotion. (2004) 18, no. 8, 1121–1133, 10.1080/02699930441000030, 2-s2.0-10944248381.

[25] Vine V. , Boyd R. L. , and Pennebaker J. W. , Natural Emotion Vocabularies as Windows on Distress and Well-Being, Nature Communications. (2020) 11, no. 1, 10.1038/s41467-020-18349-0, 4525. PMC7483527 32913209

[26] Kross E. , Verduyn P. , and Boyer M. , et al.Does Counting Emotion Words on Online Social Networks Provide a Window Into People’s Subjective Experience of Emotion? A Case Study on Facebook, Emotion. (2019) 19, no. 1, 97–107, 10.1037/emo0000416, 2-s2.0-85044871249. 29620384

[27] Sun J. , Schwartz H. A. , Son Y. , Kern M. L. , and Vazire S. , The Language of Well-Being: Tracking Fluctuations in Emotion Experience through Everyday Speech, Journal of Personality and Social Psychology. (2020) 118, no. 2, 364–387, 10.1037/pspp0000244. 30945904

[28] Seabrook E. M. , Kern M. L. , Fulcher B. D. , and Rickard N. S. , Predicting Depression From Language-Based Emotion Dynamics: Longitudinal Analysis of Facebook and Twitter Status Updates, Journal of Medical Internet Research. (2018) 20, no. 5, 10.2196/jmir.9267, 2-s2.0-85047569467, e168. 29739736 PMC5964306

[29] Beck A. T. and Bredemeier K. , A Unified Model of Depression, Clinical Psychological Science. (2016) 4, no. 4, 596–619, 10.1177/2167702616628523, 2-s2.0-84986910236.

[30] Edwards T. M. and Holtzman N. S. , A Meta-Analysis of Correlations Between Depression and First-Person Singular Pronoun use, Journal of Research in Personality. (2017) 68, 63–68, 10.1016/j.jrp.2017.02.005, 2-s2.0-85014283711.

[31] Berry-Blunt A. K. , Holtzman N. S. , Donnellan M. B. , and Mehl M. R. , The Story of “I” Tracking: Psychological Implications of Self-Referential Language use, Social and Personality Psychology Compass. (2021) 15, no. 12, 10.1111/spc3.12647.

[32] Tackman A. M. , Sbarra D. A. , and Carey A. L. , et al.Depression, Negative Emotionality, and Self-Referential Language: A Multi-Lab, Multi-Measure, and Multi-Language-Task Research Synthesis, Journal of Personality and Social Psychology. (2019) 116, no. 5, 817–834, 10.1037/pspp0000187, 2-s2.0-85042869093. 29504797

[33] Bandura A. , Theoretical Perspectives on Human Agency, 2008, Oxford University Press.

[34] Formanowicz M. , Pietraszkiewicz A. , Roessel J. , Suitner C. , Witkowska M. , and Maass A. , “Make it Happen!”, Social Psychology. (2021) 52, no. 2, 75–89, 10.1027/1864-9335/a000435.

[35] Pietraszkiewicz A. , Formanowicz M. , Gustafsson Sendén M. , Boyd R. L. , Sikström S. , and Sczesny S. , The Big Two Dictionaries: Capturing Agency and Communion in Natural Language, European Journal of Social Psychology. (2019) 49, no. 5, 871–887, 10.1002/ejsp.2561, 2-s2.0-85061908899.

[36] Nikadon J. , Suitner C. , Erseghe T. , Dzanko L. , and Formanowicz M. , BERTAgent: The Development of a Novel Tool to Quantify Agency in Textual Data, Journal of Experimental Psychology: General. (2025) 154, no. 7, 1855–1877, 10.1037/xge0001740. 40354292

[37] Boyd R. L. and Pennebaker J. W. , Language-Based Personality: A New Approach to Personality in a Digital World, Current Opinion in Behavioral Sciences. (2017) 18, 63–68, 10.1016/j.cobeha.2017.07.017, 2-s2.0-85026744838.

[38] Madera J. M. , Hebl M. R. , and Martin R. C. , Gender and Letters of Recommendation for Academia: Agentic and Communal Differences, Journal of Applied Psychology. (2009) 94, no. 6, 1591–1599, 10.1037/a0016539, 2-s2.0-72249104454. 19916666

[39] Weingarten E. , Chen Q. , McAdams M. , Yi J. , Hepler J. , and Albarracin D. , On Priming Action: Conclusions From a Meta-Analysis of the Behavioral Effects of Incidentally-Presented Words, Current Opinion in Psychology. (2016) 12, 53–57, 10.1016/j.copsyc.2016.04.015, 2-s2.0-84973442859. 27957520 PMC5147746

[40] Witkowska M. , Beneda M. , Nikadon J. , Suitner C. , Casara B. G. S. , and Formanowicz M. , Riot Like a Girl? Gender-Stereotypical Associations Boost Support for Feminist Online Campaigns, Sex Roles. (2024) 90, no. 9, 1262–1284, 10.1007/s11199-024-01502-0.

[41] Beneda M. , Kowalski J. , and Witkowska M. , Words Without Power: Reduced Semantic (but not grammatical) Agency Signals Low Mood and Self-Esteem, Journal of Affective Disorders. (2026) 400, 121170. 41558594 10.1016/j.jad.2026.121170

[42] Fasoli F. and Formanowicz M. , Can Agentic Messages Help? Linguistic Strategies to Counteract Voice-Based Sexual Orientation Discrimination, British Journal of Social Psychology. (2024) 63, no. 3, 1515–1534, 10.1111/bjso.12739. 38451067

[43] Nicolas G. , Bai X. , and Fiske S. T. , Comprehensive Stereotype Content Dictionaries Using a Semi-Automated Method, European Journal of Social Psychology. (2021) 51, no. 1, 178–196, 10.1002/ejsp.2724.

[44] Nolen-Hoeksema S. and Jackson B. , Mediators of the Gender Difference in Rumination, Psychology of Women Quarterly. (2001) 25, no. 1, 37–47, 10.1111/1471-6402.00005, 2-s2.0-0035285210.

[45] Kessler R. C. , Epidemiology of Women and Depression, Journal of Affective Disorders. (2003) 74, no. 1, 5–13, 10.1016/S0165-0327(02)00426-3, 2-s2.0-0037341830. 12646294

[46] Shorey S. , Chee C. Y. I. , Ng E. D. , Chan Y. H. , San Tam W. W. , and Chong Y. S. , Prevalence and Incidence of Postpartum Depression Among Healthy Mothers: A Systematic Review and Meta-Analysis, Journal of Psychiatric Research. (2018) 104, 235–248, 10.1016/j.jpsychires.2018.08.001, 2-s2.0-85051407842. 30114665

[47] O’Hara M. W. and McCabe J. E. , Postpartum Depression: Current Status and Future Directions, Annual Review of Clinical Psychology. (2013) 9, no. 1, 379–407, 10.1146/annurev-clinpsy-050212-185612, 2-s2.0-84875883757. 23394227

[48] Patel M. , Bailey R. K. , Jabeen S. , Ali S. , Barker N. C. , and Osiezagha K. , Postpartum Depression: A Review, Journal of Health Care for the Poor and Underserved. (2012) 23, no. 2, 534–542, 10.1353/hpu.2012.0037, 2-s2.0-84860449009. 22643605

[49] Kantrowitz-Gordon I. , Internet Confessions of Postpartum Depression, Issues in Mental Health Nursing. (2013) 34, no. 12, 874–882, 10.3109/01612840.2013.806618, 2-s2.0-84889021746. 24274243

[50] Moore D. , Drey N. , and Ayers S. , A Meta-Synthesis of Women’s Experiences of Online Forums for Maternal Mental Illness and Stigma, Archives of Women’s Mental Health. (2020) 23, no. 4, 507–515, 10.1007/s00737-019-01002-1. 31646392

[51] Meyling M. M. G. , Frieling M. E. , Vervoort J. P. M. , Feijen-de Jong E. I. , and Jansen D. E. M. C. , Health Problems Experienced by Women During the First Year Postpartum: A Systematic Review, European Journal of Midwifery. (2023) 7, 10.18332/ejm/173417, 42. 38111746 PMC10726257

[52] Myers S. , Sharma A. , Gupta P. , and Lin J. , Information Network or Social Network? The Structure of the Twitter Follow Graph, *Companion Proceedings of the 23rd International Conference on World Wide Web (WWW ’14 Companion)*, 2014, Association for Computing Machinery (ACM), 493–498, 10.1145/2567948.

[53] Yahya N. H. and Rahim H. A. , Linguistic Markers of Depression: Insights From English-Language Tweets Before and During the COVID-19 Pandemic, Language and Health. (2023) 1, no. 2, 36–50, 10.1016/j.laheal.2023.10.001.

[54] Kim J. , Uddin Z. A. , and Lee Y. , et al.A Systematic Review of the Validity of Screening Depression Through Facebook, Twitter, Instagram, and Snapchat, Journal of Affective Disorders. (2021) 286, 360–369, 10.1016/j.jad.2020.08.091. 33691948

[55] Di Cara N. H. , Maggio V. , Davis O. S. P. , and Haworth C. M. A. , Methodologies for Monitoring Mental Health on Twitter: Systematic Review, Journal of Medical Internet Research. (2023) 25, 10.2196/42734, e42734. 37155236 PMC10203928

[56] Boyd R. L. , Hai-Jew S. , Psychological Text Analysis in the Digital Humanities, Data Analytics in Digital Humanities, 2017, Springer, 161–189, 10.1007/978-3-319-54499-1_7.

[57] Kwon S. and Park A. , Understanding User Responses to the COVID-19 Pandemic on Twitter From a Terror Management Theory Perspective: Cultural Differences Among the US, UK, and India, Computers in Human Behavior. (2022) 128, 10.1016/j.chb.2021.107087, 107087. 34744298 PMC8558263

[58] Cohen J. , Statistical Power Analysis for the Behavioral Sciences, 1988, 2nd edition, Lawrence Erlbaum Associates.

[59] Grootendorst M. , BERTopic: Neural Topic Modeling With a Class-Based TF-IDF Procedure, 2022, *arXiv*:2203.05794 10.48550/arXiv.2203.05794.

[60] Devlin J. , Chang M. W. , Lee K. , and Toutanova K. , BERT: Pre-Training of Deep Bidirectional Transformers for Language Understanding, 2019, 10.48550/arXiv.1810.04805, 1810.04805.

[61] Rahutomo F. , Kitasuka T. , and Aritsugi M. , Semantic Cosine Similarity, 4, *The 7th International Student Conference on Advanced Science and Technology ICAST*, 2012, Kumamoto, Japan, International Student Conference on Advanced Science and Technology (ICAST).

[62] Pennebaker J. W. , Francis M. E. , and Booth R. J. , Linguistic Inquiry and Word Count: LIWC 2001, 2001, Lawrence Erlbaum Associates.

[63] Zimmermann J. , Wolf M. , Bock A. , Peham D. , and Benecke C. , The Way We Refer to Ourselves Reflects How We Relate to Others: Associations between First-Person Pronoun use and Interpersonal Problems, Journal of Research in Personality. (2013) 47, no. 3, 218–225, 10.1016/j.jrp.2013.01.008, 2-s2.0-84874442916.

[64] Boyd R. L. , Ashokkumar A. , Seraj S. , and Pennebaker J. W. , The Development and Psychometric Properties of LIWC-22, 2022, University of Texas at Austin, https://www.liwc.app.

[65] Kroenke K. , Spitzer R. L. , and Williams J. B. W. , The Patient Health Questionnaire-2: Validity of a Two-Item Depression Screener, Medical Care. (2003) 41, no. 11, 1284–1292, 10.1097/01.MLR.0000093487.78664.3C, 2-s2.0-0642311548. 14583691

[66] Bathina K. C. , Ten Thij M. , Lorenzo-Luaces L. , Rutter L. A. , and Bollen J. , Individuals With Depression Express More Distorted Thinking on Social Media, Nature Human Behaviour. (2021) 5, no. 4, 458–466, 10.1038/s41562-021-01050-7. 33574604

[67] Zhang Y. , Lyu H. , Liu Y. , Zhang X. , Wang Y. , and Luo J. , Monitoring Depression Trends on Twitter During the COVID-19 Pandemic: Observational Study, JMIR Infodemiology. (2021) 1, no. 1, 10.2196/26769, e26769. 34458682 PMC8330892

[68] Huson L. W. , Performance of Some Correlation Coefficients When Applied to Zero-Clustered Data, Journal of Modern Applied Statistical Methods. (2007) 6, no. 2, 530–536, 10.22237/jmasm/1193890560, 2-s2.0-56249108064.

[69] Lumley T. , Analysis of Complex Survey Samples, Journal of Statistical Software. (2004) 9, no. 8, 1–19, 10.18637/jss.v009.i08.

[70] Zhu Y. Q. and Chen H. G. , Social Media and Human Need Satisfaction: Implications for Social Media Marketing, Business Horizons. (2015) 58, no. 3, 335–345, 10.1016/j.bushor.2015.01.006, 2-s2.0-84928824231.

[71] Kwon S. and Park A. , Examining Thematic and Emotional Differences across Twitter, Reddit, and YouTube: The Case of COVID-19 Vaccine Side Effects, Computers in Human Behavior. (2023) 144, 10.1016/j.chb.2023.107734, 107734. 36942128 PMC10016349

[72] Theocharis Y. , Cardenal A. S. , and Jin S. , et al.Does the Platform Matter? Social Media and COVID-19 Conspiracy Theory Beliefs in 17 Countries, New Media and Society. (2023) 25, no. 12, 3412–3437, 10.1177/14614448211045666.

[73] Jiang H. , Zhou R. , Zhang L. , Wang H. , and Zhang Y. , Sentence-Level Topic Models for Associated Topics Extraction, World Wide Web-Internet and Web Information Systems. (2019) 22, no. 6, 2545–2560, 10.1007/s11280-018-0639-1, 2-s2.0-85055571703.

[74] Manikonda L. , Beigi G. , Liu H. , and Kambhampati S. , Twitter for Sparking a Movement, Reddit for Sharing the Moment: #MeToo Through the Lens of Social Media, 2018, 10.48550/arXiv.1803.08022, 1803.08022.

[75] Rohde J. A. , Sibley A. L. , and Noar S. M. , Topics Analysis of Reddit and Twitter Posts Discussing Inflammatory Bowel Disease and Distress From 2017 to 2019, Crohn’s and Colitis 360. (2021) 3, no. 3, 10.1093/crocol/otab044, otab044. PMC9802272 36776642

[76] Sik D. , Németh R. , and Katona E. , Topic Modelling Online Depression Forums: Beyond Narratives of Self-Objectification and Self-Blaming, Journal of Mental Health. (2023) 32, no. 2, 386–395, 10.1080/09638237.2021.1979493. 34582309

[77] Ziemer K. S. and Korkmaz G. , Using Text to Predict Psychological and Physical Health: A Comparison of Human Raters and Computerized Text Analysis, Computers in Human Behavior. (2017) 76, 122–127, 10.1016/j.chb.2017.06.038, 2-s2.0-85024125398.

[78] Sierra G. , Andrade-Palos P. , and Bel-Enguix G. , et al.Suicide Risk Factors: A Language Analysis Approach in Social Media, Journal of Language and Social Psychology. (2022) 41, no. 3, 312–330, 10.1177/0261927X211036171.

[79] Matero M. , Idnani A. , and Son Y. , et al.Suicide Risk Assessment With Multi-Level Dual-Context Language and BERT, *Proceedings of the Fifth Workshop on Computational Linguistics and Clinical Psychology: From Keyboard to Clinic*, 2019, Association for Computational Linguistics, 39–44, 10.18653/v1/W19-3005.

[80] Memon A. M. , Sharma S. G. , Mohite S. S. , and Jain S. , The Role of Online Social Networking on Deliberate Self-Harm and Suicidality in Adolescents: A Systematized Review of Literature, Indian Journal of Psychiatry. (2018) 60, no. 4, 384–392, 10.4103/psychiatry.IndianJPsychiatry_414_17, 2-s2.0-85058699087. 30581202 PMC6278213

[81] Batt M. M. , Duffy K. A. , Novick A. M. , Metcalf C. A. , and Epperson C. N. , Is Postpartum Depression Different From Depression Occurring Outside of the Perinatal Period? A Review of the Evidence, Focus. (2020) 18, no. 2, 106–119, 10.1176/appi.focus.20190045. 33162848 PMC7587887

[82] Kramer M. D. , Krueger R. F. , and Hicks B. M. , The Role of Internalizing and Externalizing Liability Factors in Accounting for Gender Differences in the Prevalence of Common Psychopathological Syndromes, Psychological Medicine. (2008) 38, no. 1, 51–61, 10.1017/S0033291707001572, 2-s2.0-37249006210. 17892625

