# Supplementary material for: Semantic Agency Patterns Signal Depressive Experiences: Evidence From Postpartum Communication on Social Media
Source: Depress Anxiety. 2026 Feb 8;2026:6485997. doi: 10.1155/da/6485997 (PMC12883671; doi:10.1155/da/6485997)
Supplement: Supplementary file 1 — Supporting Information Distributional properties of the linguistic variables used in the correlation analyses are reported in the Supplementary Online Materials (Figures S1–S3 for Study 1; Figures S4–S6 for Study 2). Semantic agency showed an approximately symmetric, near‐normal distribution, whereas word‐count measures were highly skewed and zero‐inflated. To account for these distributional characteristics, we conducted robustness checks using Spearman rank‐order correlations with cluster‐robust standard errors; results were highly consistent with Pearson correlations (Tables S1–S2). [file DA-2026-6485997-s001.docx]

**Semantic Agency Patterns Signal Depressive Experiences: Evidence from Postpartum Communication on Social Media**

**Supplementary Online Materials**

To evaluate distributional characteristics relevant for the correlation analyses, we examined the distributions of semantic agency, first-person singular pronouns, and positive and negative emotion words (see Figures S1-S3 for Study 1 and S4-S6 for Study 2). The plots reveal that semantic agency is approximately symmetric and near-normal, whereas the word count measures are highly skewed and zero-inflated, with most observations clustered at or near zero and only a minority of posts showing higher frequencies. These deviations from normality highlight the need to conduct robustness checks. Accordingly, we computed Spearman rank-order correlations with cluster-robust standard errors, which yielded results highly consistent with those obtained using Pearson correlations (see Table S1 and Table S2). This convergence across methods provides additional evidence for the robustness of our findings.

**Study 1 (Twitter)**

**Figure S1**

*Histograms of Semantic Agency, I-Words, Positive Emotion Words, and Negative Emotion Words*


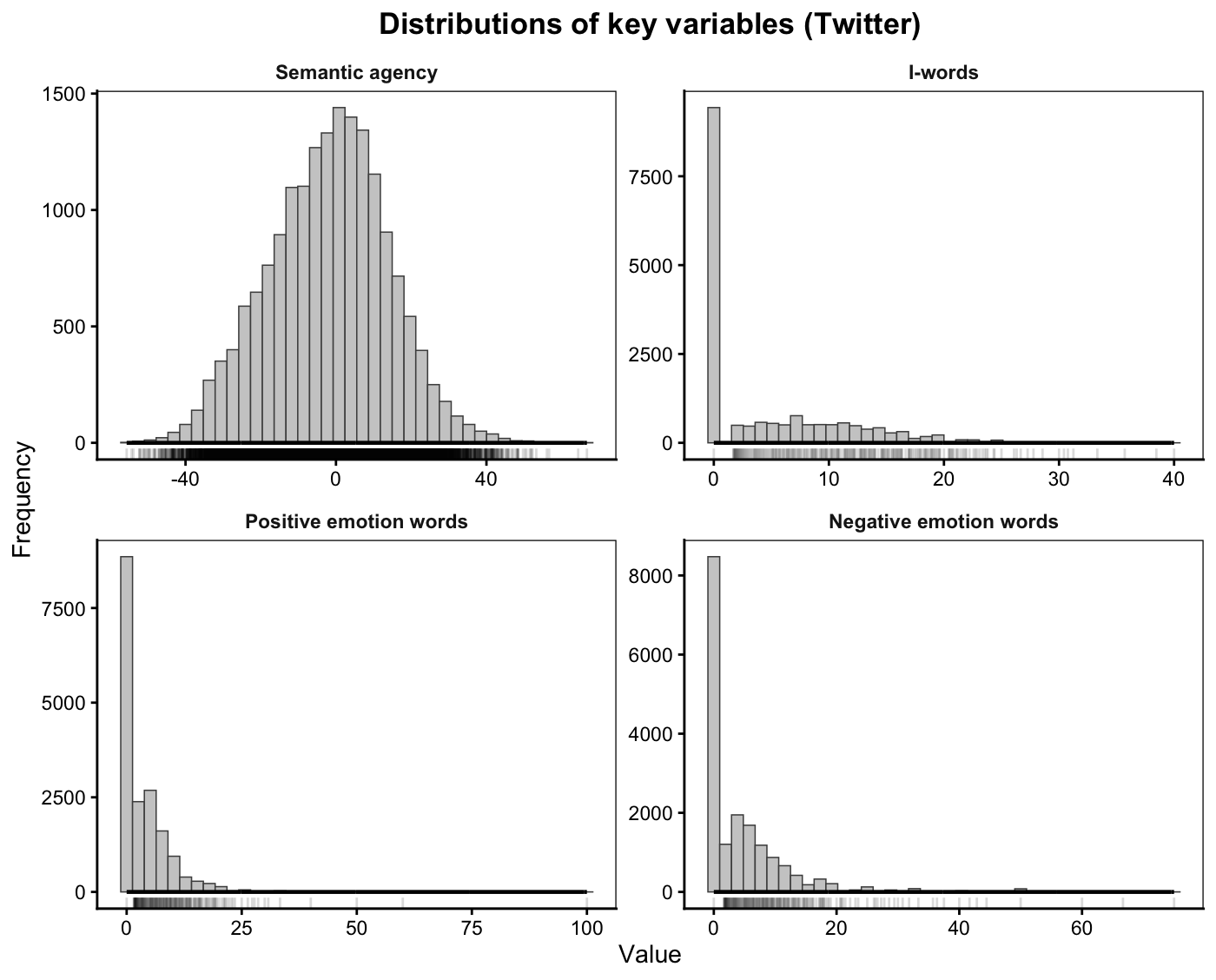


**Figure S2**

*Q–Q Plots for Semantic Agency, I-Words, Positive Emotion Words, and Negative Emotion Words*


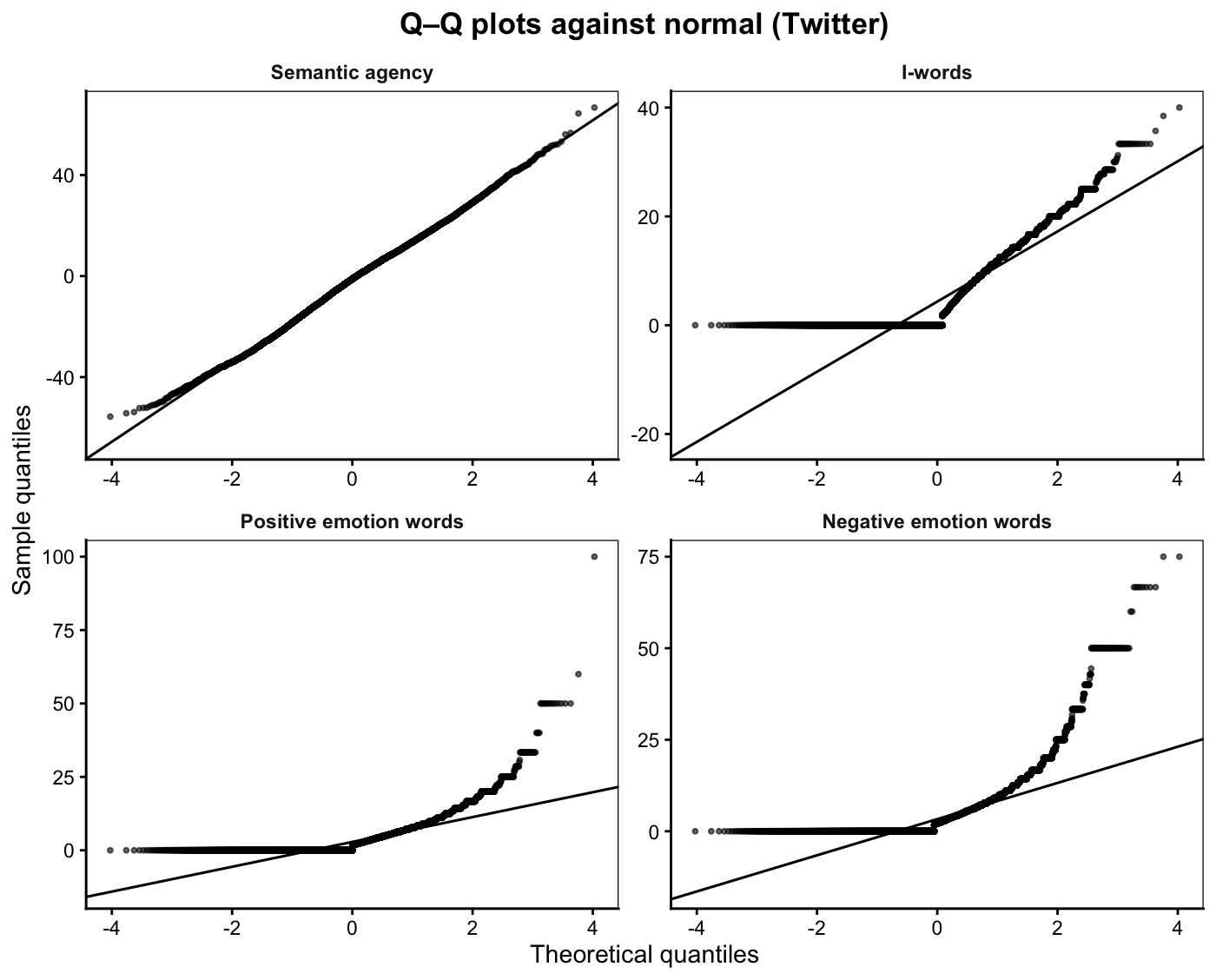


**Figure S3**

*Violin Plots for Semantic Agency, I-Words, Positive Emotion Words, and Negative Emotion Words*


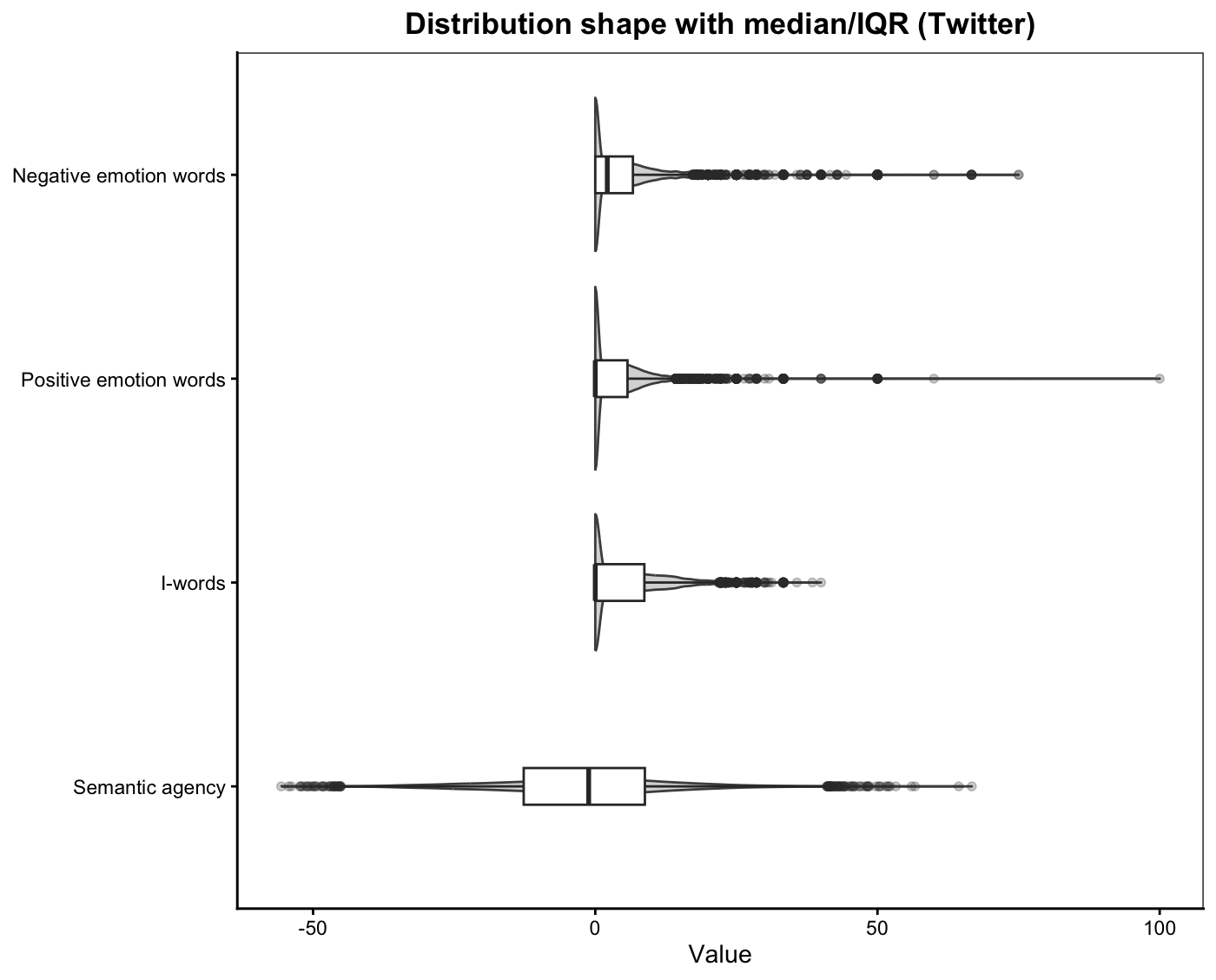


**Table S1**

*Pearson and Spearman Correlations in Study 1*

|  | 1. | 2. | 3. |
| --- | --- | --- | --- |
| 1. Semantic agency |  |  |  |
| 2. I-words | -.12***/-.14*** |  |  |
| 3. Positive emotion words | .23***/.26*** | -.04***/.02 |  |
| 4. Negative emotion words | -.43***/-.47*** | -.02**/.05*** | -.13***/-.13*** |

*Note.* Entries are Pearson / Spearman correlations; cluster-robust SE, authors as clusters; p-values were adjusted using the Holm–Bonferroni method within each family of tests; ** *p* < .01; *** *p* < .001.

**Study 2 (Reddit)**

**Figure S4**

*Histograms of Semantic Agency, I-Words, Positive Emotion Words, and Negative Emotion Words*


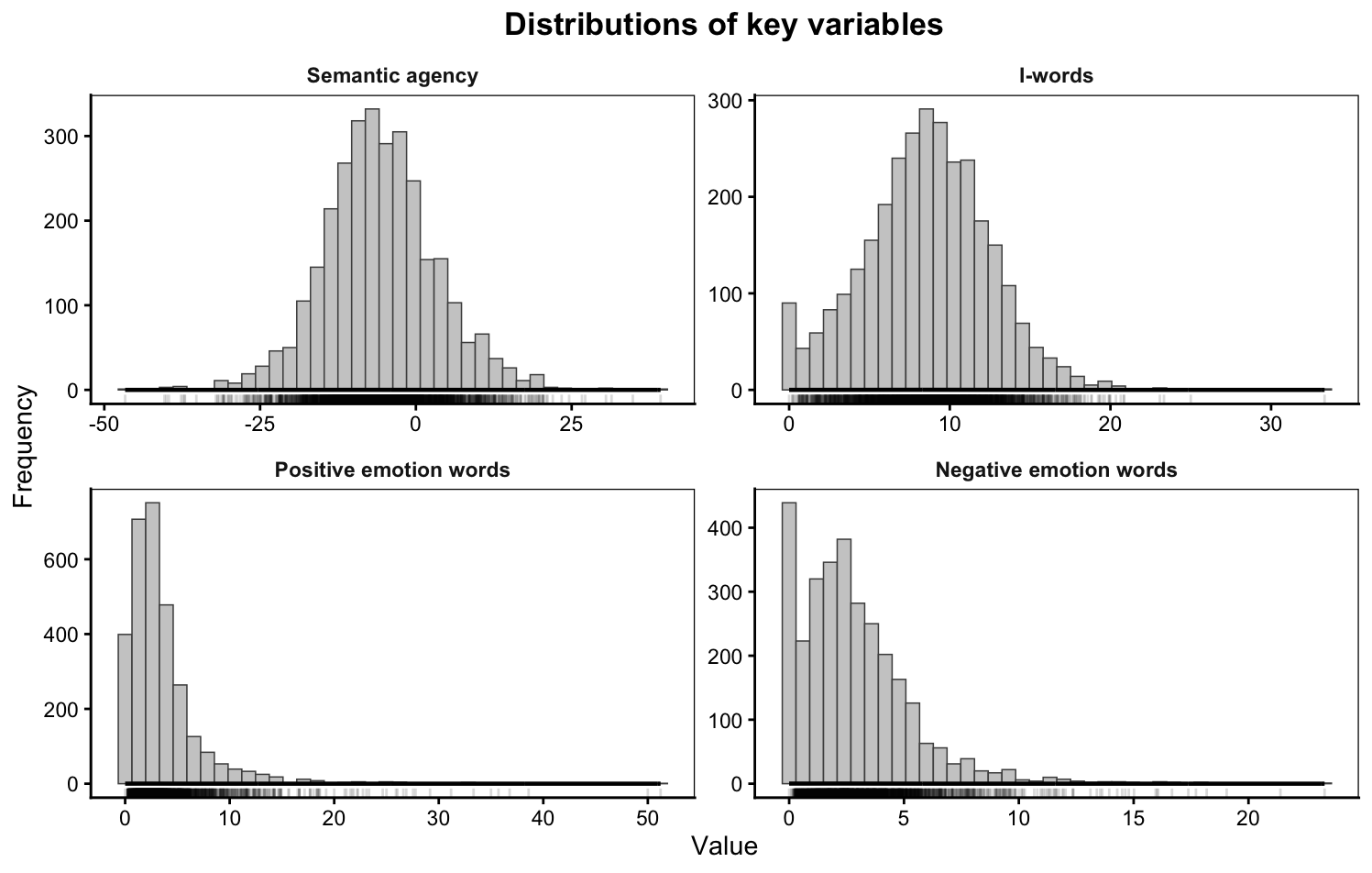


**Figure S5**

*Q–Q plots for Semantic Agency, I-Words, Positive Emotion Words, and Negative Emotion Words*


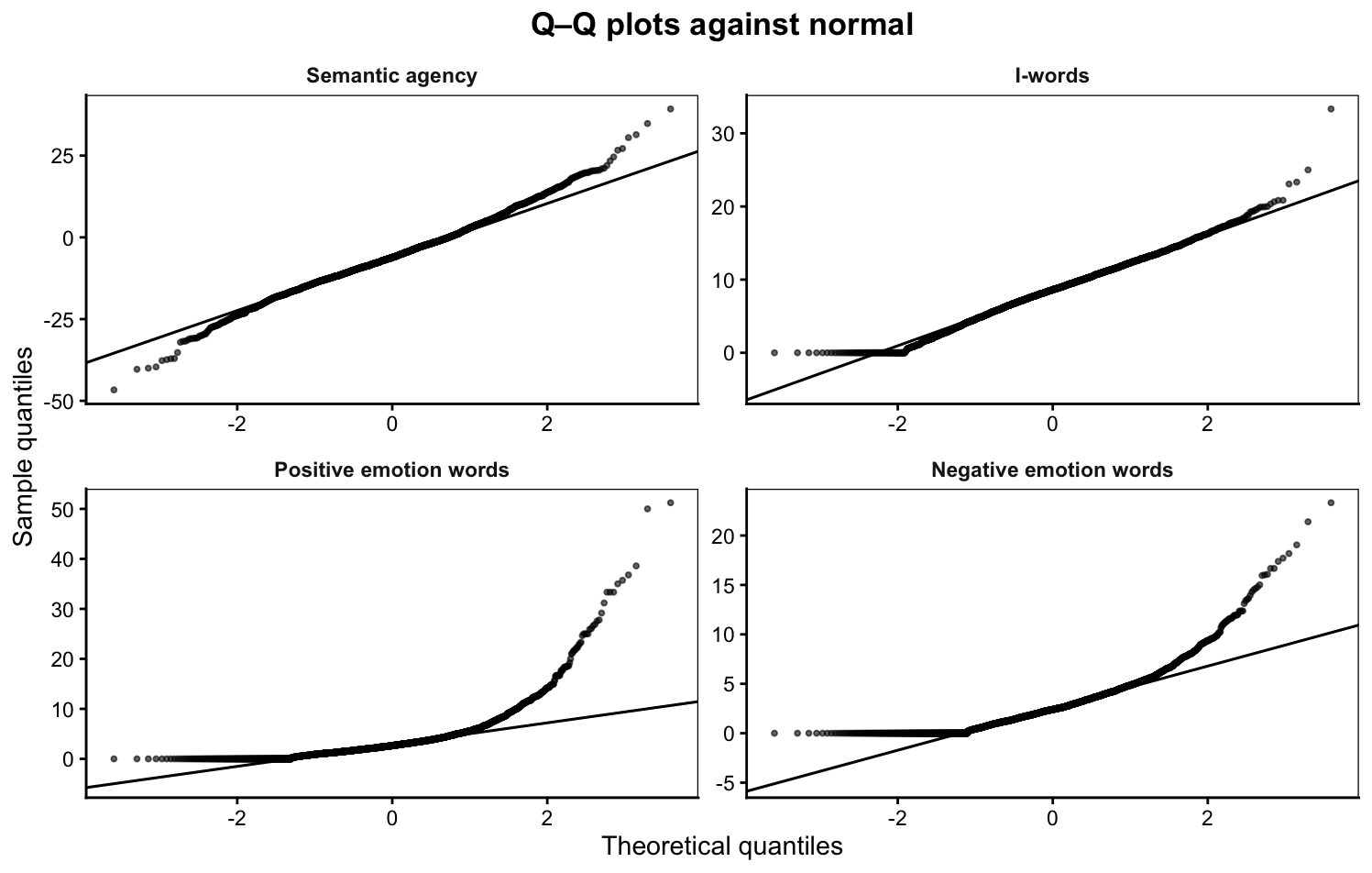


**Figure S6**

*Violin Plots for Semantic Agency, I-Words, Positive Emotion Words, and Negative Emotion Words*


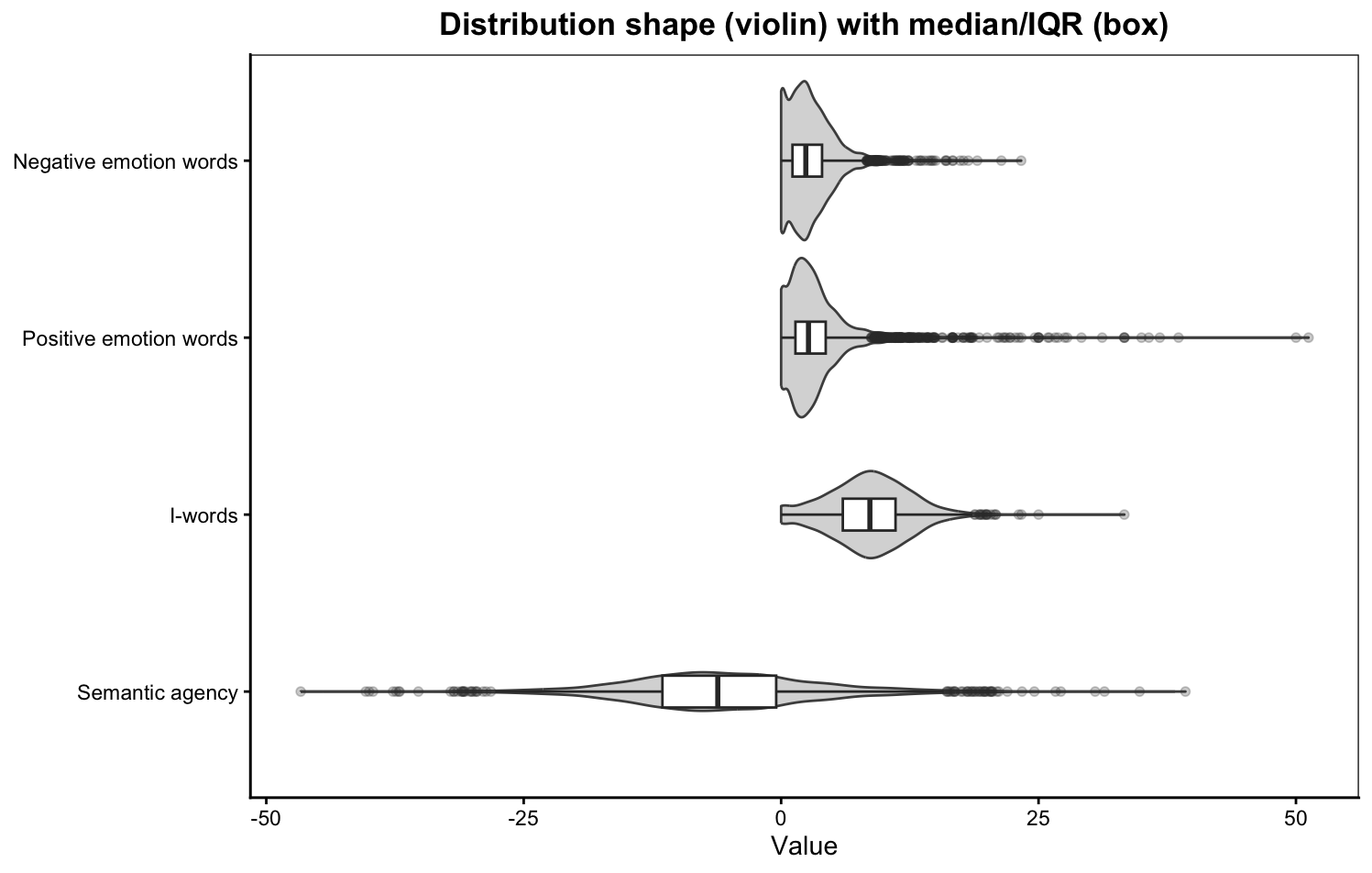


**Table S2.**

*Pearson and Spearman Correlations in Study 2*

|  | 1. | 2. | 3. |
| --- | --- | --- | --- |
| 1. Semantic agency |  |  |  |
| 2. I-words | -.34***/-.34*** |  |  |
| 3. Positive emotion words | .26***/.26*** | -.15***/-.12*** |  |
| 4. Negative emotion words | -.48***/-.53*** | .19***/.25*** | -.14***/-.10*** |

*Note.* Entries are Pearson / Spearman correlations; cluster-robust SE, authors as clusters; p-values were adjusted using the Holm–Bonferroni method within each family of tests; *** *p* < .001.
